# A Digital Platform for Facilitating Personalized Dementia Care in Nursing Homes: Formative Evaluation Study

**DOI:** 10.2196/25705

**Published:** 2021-05-28

**Authors:** Gubing Wang, Armagan Albayrak, Gerd Kortuem, Tischa JM van der Cammen

**Affiliations:** 1 Faculty of Industrial Design Engineering Delft University of Technology Delft Netherlands

**Keywords:** human-centered design, data visualizations, person-centered care, people with dementia, assistive technology, health care design, care management, internet of things, data-driven design, data-enabled design

## Abstract

**Background:**

Care personalization is key to the well-being of people with dementia according to person-centered care. With the development of the internet of things, a large quantity of personal data can be collected securely and reliably, which has the potential to facilitate care personalization for people with dementia. Yet, there are limited assistive technologies developed for this purpose, and the user acceptance of assistive technologies is low in nursing homes. Therefore, through a data-enabled design approach, a digital platform was developed for helping the care team in a nursing home to personalize dementia care, specifically in the management of behavioral and psychological dementia symptoms.

**Objective:**

This study aimed to evaluate the digital platform in a real-life context with potential users from the following two aspects: (1) to explore if the digital platform could help with generating insights on the current state of each person with dementia and (2) to gather feedback on the digital platform from the care team.

**Methods:**

The digital platform was deployed in the nursing home for 7 weeks and the data collected were visualized and presented to the care team via the digital platform. The visualizations were analyzed by the researchers for pattern detection. Meanwhile, the care team was asked to examine the visualizations and were interviewed for the following: (1) if any insights and actions were generated from the examination, (2) the usefulness of the digital platform, and (3) the improvements they would like to see.

**Results:**

The data collected on the digital platform demonstrated its potential for pattern detection. Insights were generated by the care team and categorized into “client level,” “ward level,” and “team level.” The corresponding actions taken by the care team were classified into “investigation” and “implementation.” User acceptance varied across the care team, and three aspects of improvement for the digital platform were identified.

**Conclusions:**

By evaluating the digital platform, this study gained insights on applying data-enabled design for personalizing dementia care; besides, it offers future researchers some recommendations on how to integrate assistive technologies in the nursing home context.

## Introduction

### Background

Dementia has physical, psychological, and social impacts on affected people, their caregivers, and their families, and it is an economic burden to the society at large [1]. The “person-centered” perspective on dementia care was introduced in care practice by Kitwood [2], where “in addition to neurological impairments, the personality, past experiences, health, and other aspects of the person with dementia also influence how the person will behave.” Inspired by Kitwood’s theories, researchers in dementia care have been exploring how to manage Behavioral and Psychological Symptoms of Dementia (BPSD) in a person-centered manner [3-6].

Over 80% of people with dementia will develop BPSD during the course of their disease [7], and 90% of people with dementia living in nursing homes exhibit BPSD [8]. BPSD contribute to the most stressful, complex, and costly aspects of dementia care, which commonly result in poor health outcomes for people with dementia [9]. Personalization is a key element of the person-centered care approach [10], and most of the current practices accommodate the past experiences, hobbies, capabilities, and preferences of people with dementia in developing personalized care plans [11,12]. However, some of these capabilities, behaviors, personalities, and preferences of people with dementia could change over time as dementia progresses [13]. We propose that these changes should be monitored and considered for providing a holistic approach to personalized BPSD management. We argue a personalized care plan can only be helpful when it is up to date. Yet, it takes time and effort for care teams to notice and adapt to some of these changes given their high workload [14]. Recent research has been investigating the potential of data-driven assistive technologies in care personalization.

### Related Work

Over the years, low-cost sensors, wearables, electronic health records, and artificial intelligence have been deployed in the health care setting to collect and analyze data about an individual in terms of his/her physical condition, living environment, lifestyle choices, etc [15]. Researchers hypothesize that these data could help health care professionals to make better and more timely decisions for each patient and hence allow the care received by the patient to be personalized and continuous rather than general and episodic [16]. Collectively, data-driven care personalization is made possible by assistive technologies that can integrate the data and best care practices for health service delivery [17]. A global agenda for personalized telehealth has been proposed [18].

Previous attempts have explored the application of data-driven assistive technologies in the field of cancer care [19], physiotherapy [20], cardiovascular disease [21], and elderly care at home [22]. Conversational agents [23] and data visualizations [19] are being developed for facilitating the interactions between the technologies and their users. However, few studies have investigated this type of assistive technology in personalizing dementia care. The adoption rate of assistive technologies for dementia care in nursing homes has been low. A scenario-based survey study found that caregivers think assistive technologies have not been tailored to their needs and concerns [24]. Similarly, a systematic review identified that the acceptance of assistive technologies is low without users’ input during its development process [25]. Therefore, a main reason for the low adoption has been identified to be the lack of understanding regarding the interactions between the technology and the people involved.

### Research Context

In our prior work, we involved the care team from a Dutch nursing home and developed a digital platform for personalizing BPSD management via a data-enabled design approach. Data-enabled design is about using quantitative data from sensors and qualitative data from users in the field as creative design material to inspire and inform the design process [26]. These data can be highly personalized for an individual. We, therefore, hypothesize that data-enabled design offers an approach to transform these personal data into valuable up-to-date insights about each person with dementia for the care team and hence can facilitate the care team in personalizing BPSD management holistically. The digital platform was then developed for visualizing and presenting the combination of quantitative and qualitative data to the care team and was evaluated in the ward for 15 days. A detailed description of this digital platform can be found in a previous report by Wang et al [27].

We positioned this digital platform as an assistive technology to facilitate the care team in personalizing BPSD management. Studies from the perspectives of care research [28,29], anthropology [30], and gerontology [31] have pointed out that understanding the interplay between the technology and the context (eg, users) to which it is introduced early on could help in the integration of the technology for regular use in the future. Therefore, this study was conducted to explore the effects of the digital platform as an assistive technology on the facilitation of personalized BPSD management in the daily care practice in a nursing home for a longer period of time. This is a qualitative study with the focus of researching the user experience of a care team in using the digital platform.

### Aims

This study aimed to (1) explore if the data visualizations of the digital platform could help with generating insights into the current state of each person with dementia and (2) gather feedback on the digital platform from a care team after deployment in a nursing home for a longer period of time. In this way, it builds on our preceding work by investigating the interplay of the digital platform and the care team in a longer time frame to explore the opportunities and challenges for personalizing BPSD care with assistive technologies in a real-life context among potential users.

## Methods

### Study Setup

In our prior study, an Indoor Positioning System (IPS) was deployed in one of the nursing home sites of Zorggroep Elde Maasduinen in the Netherlands to collect continuous location data from the residents and the professional caregivers (hereafter the caregivers). Zorggroep Elde Maasduinen is a large-scale organization for the care of older adults in the south of the Netherlands [32], with multiple sites throughout the south of the Netherlands. This study was performed in a nursing-home site in Boxtel, which has a special ward (the Oleander) for caring for residents exhibiting BPSD. This ward is a specific ward for assessing the behaviors of people in the later stages of dementia (from moderate to late stages), who exhibit BPSD, with the aim to find an intervention for these symptoms for each individual person with dementia so that, in time, the person with dementia can return to one of the regular dementia wards in the nursing home.

The Oleander was selected to be the development site of the digital platform since it offers a typical case of BPSD management in an institutional setting. The IPS was hence deployed in the Oleander with the consent of all the legal representatives of all the residents and all care team members. Contextual information concerning the culture of the nursing home, the ward ambiance of the Oleander, and the working structure of the care team is provided in Multimedia Appendix 1. There were 10 residents living in this ward, and the care team regarded each resident as a unique individual. In order to understand the user experiences of the residents and the care team with the use of the digital platform in-depth, the minimum sample size for this study was three people with dementia (given our budget limit).

The study protocol was approved by the Human Research Ethics Committee of Delft University of Technology and the Board of Directors of Zorggroep Elde Maasduinen (see Multimedia Appendix 2 for the ethics approval letters of Delft University of Technology and Zorggroep Elde Maasduinen). Since this study involved collecting location data from people with dementia and caregivers, written informed consent was obtained from the caregivers and from the legal representatives of the people with dementia.

Location data are collected because, among all types of sensor data, this type has been used for monitoring BPSD and is recognized as central to the context of BPSD management [33]. Specifically, not only movement patterns but also other relevant parameters (eg, traveled distance and interaction time with others) could be derived from location data. Both caregivers and residents who participated were given tags since the digital platform was designed with the intention to record the interaction times between caregivers and people with dementia. This interaction time was defined as when and for how long caregivers interacted with people with dementia per day. We programmed the IPS so that a physical distance between two tags of less than half a meter for more than 1 minute was registered as an interaction.

**Figure 1:**
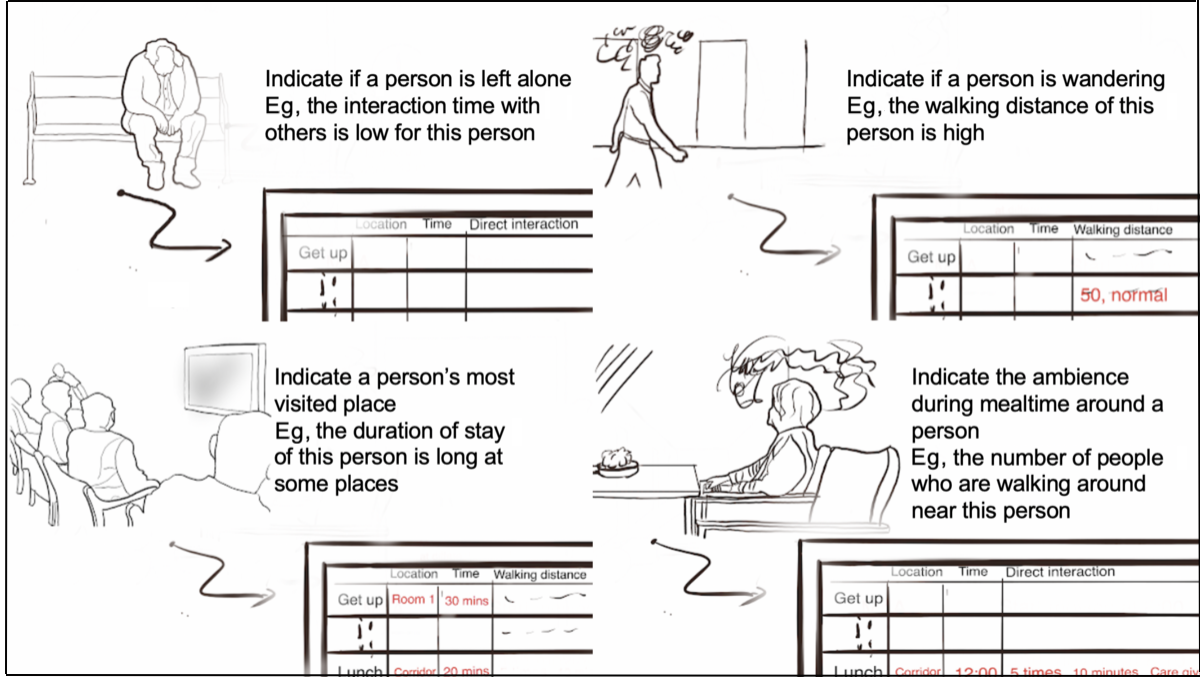
Four envisioned scenarios where the digital platform could help with personalized BPSD (Behavioral and Psychological Symptoms of Dementia) management.

For each resident, several other parameters specific to the daily routine and behavior of the resident were derived from the location data collected (eg, traveled distance, traveled trajectory, and duration of stay). Meanwhile, qualitative data about each resident (eg, daily report) were collected to contextualize the location data. How might the digital platform help with personalized BPSD management is illustrated with a few envisioned scenarios in Figure 1.

### Study Design

**Figure 2:**
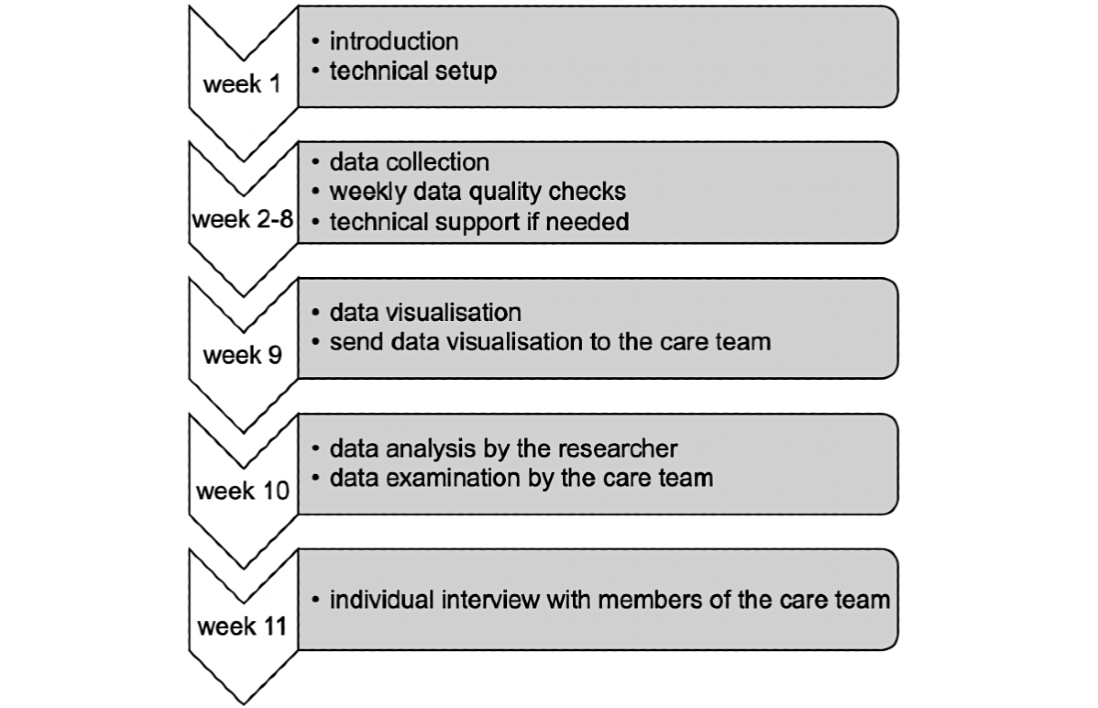
Study procedure.

In this study, a longitudinal design was employed. As demonstrated in the previous section, evaluating the digital platform for BPSD management in the Oleander constituted a macro case study. Within the macro case study were nested micro case studies, and each micro case study was about one person with dementia. The sampling strategy of this study was a combination of typical case sampling and criterion sampling. Regarding typical case sampling, a broad consensus about what is typical was achieved via a discussion involving the research team and care team. All 10 residents in the Oleander were identified as exhibiting typical BPSD. Within these residents, the legal representatives of eight residents with dementia signed the consent form. Three residents showed signs of dislike toward the tags, and they were excluded from the study. Two participants dropped out later because of signs of dislike toward the tags. Thus, three participants completed the study, resulting in three micro case studies. The three people with dementia were two females and one male. Moreover, one had vascular dementia, one had Lewy body dementia, and one had Alzheimer disease. Based on the information from the care team, all three people with dementia were in the later stages of dementia, as supported by their Mini-Mental State Examination scores. Basic information about these three people with dementia can be found in Multimedia Appendix 3. As for criterion sampling, having a direct influence on the care plan of each person with dementia was considered to be the inclusion criterion. For each person with dementia, the responsible caregiver of the person with dementia, the ward doctor (hereafter doctor), the ward psychologist (hereafter psychologist), and the ward dietitian (hereafter dietitian) had direct influences on the care plan. In addition, the ward manager (hereafter manager) was considered to have a direct influence on the care plans because of participation in multidisciplinary meetings for care plan updates. All members of the care team who met the criterion were approached, and all of them consented to participate in the study. Therefore, each micro case study included the person with dementia and his/her responsible caregiver, doctor, psychologist, dietitian, and manager. The sample size was considered to be adequate for this constructivist qualitative research, where a new, deep, and nuanced understanding was aimed to be gained on personalizing BPSD management with a data-enabled design [34]. The study procedure is illustrated in Figure 2.

Specifically, in week 1, an introduction was given to the participants, and the IPS was tested for its functionality. In weeks 2 to 8, the residents and caregivers wore tags for collecting their location data in the ward in everyday life. The caregivers wrote daily reports and gave a color code on the perceived stress levels of the people with dementia (ie, stress rating). For minimizing the workload of the care team, these tasks were designed to be as close as possible to the working routine of the care team. As for the daily report, it was part of the working routine of the care team. The daily report usually recorded when and where the person with dementia got stressed, what did he/she do, and what did the caregiver do to reduce his/her stress. Once a report was written, caregivers could select a category for the report to be uploaded to the system (eg, physical health and mental health). These reports were shared with other members of the care team, which could sometimes result in additional remarks and editing. The stress rating was done with the Crisis Development model (Crisisontwikkelingsmodel in Dutch) [35], which is a standardized assessment tool used in several Dutch nursing homes for rating the stress levels of the residents, and each member of the care team was trained to use it. This model divided the stress level of a person into green, yellow, orange, and red, indicating no stress (green) to high stress (red). The care team recorded and categorized the effective measures carried out to help a resident relax at different stress levels on a so-called signal plan. Each resident had a personalized signal plan. Based on the signal plan, the behaviors of the residents acted as signals for their stress levels. The care team could then react by adjusting the interaction style and environment for each resident according to the signal plan to reduce the stress for the resident. This way of working has been found to be helpful by the care team. Before the development of the digital platform, the care team did not explicitly record the stress of a resident over time. During this study, the caregivers were instructed to conduct the stress rating. The frequency of stress rating changed to every half an hour in this study (the previous frequency was once per day [27]) as the caregivers found that the stress levels of people with dementia usually change drastically over the day. The quality of data was checked weekly, and technical support was provided when needed.

In week 9, both quantitative and qualitative data were visualized on the digital platform. Since this study spans a longer period of time, new types of visualizations were created on the digital platform for conveying the large amount of collected data effectively. Once the visualizations were done, a notification was sent to the caregivers to start the data examination. This is because the user scenario adopted was as follows: “the responsible caregiver examines the visualizations and discusses his/her findings with other team members in a care plan meeting” (refined based on a previous study [27]). Hence, the data visualizations were developed for caregivers to do the first round of data examination, and feedback on the digital platform from the whole care team was gathered. The term “examine” was used in referring to the caregiver’s and care team’s inspection of the data so as to reserve the term “analyze” for the researchers and algorithms. In week 10, both the researchers and care team studied the visualizations with different goals. While the goal of the care team was to uncover insights about people with dementia via a combination of quantitative and qualitative data, the goal of researchers was to investigate if behavioral patterns of people with dementia could be identified with the quantitative data (ie, the availability and utility of the data) and check with the care team about the identified patterns afterward. In week 11, individual interviews were carried out with the responsible caregiver of each person with dementia, doctor, psychologist, dietitian, and manager. The doctor, psychologist, dietitian, and manager were interviewed three times, and each time, they were interviewed specifically about each individual person with dementia. This led to 15 interviews (ie, five interviews per person with dementia). The participants involved in the study are shown in Table 1.

**Table 1:** Participants involved in the study.

Study phase and participant type		Number of participants
**Data collection**		
	People with dementia	3
	Caregivers	12
**Data examination**		
	Caregivers	3
	Doctor	1
	Psychologist	1
	Dietitian	1
	Manager	1

### Data Collection

**Figure 3:**
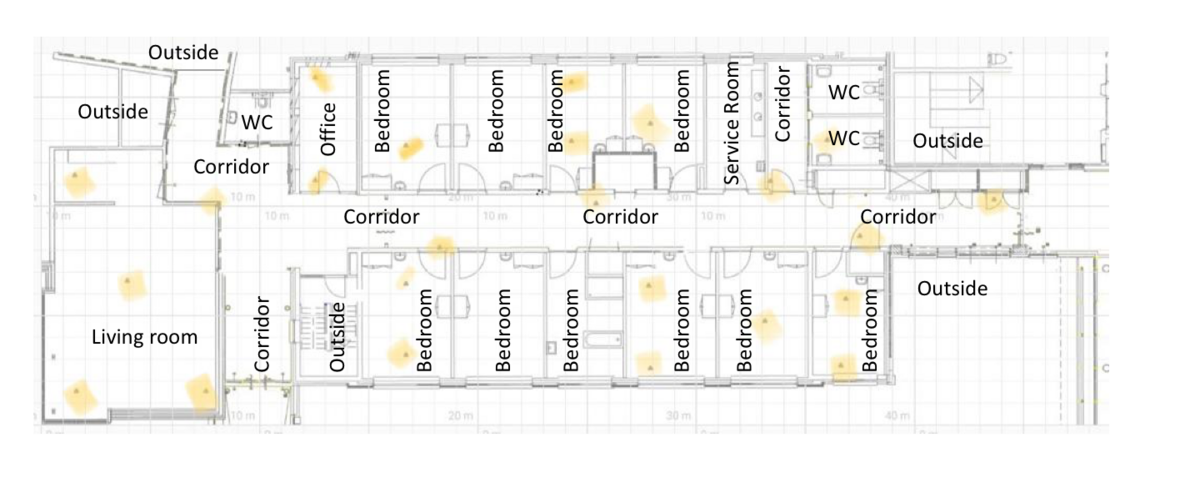
Set-up of location data collection in the ward (locations of the sensors are marked yellow on the map, and the data collected are sent to the server in the office via Wi-Fi gateways).

The categories of data collected and their collection times in this study are summarized in Table 2. Precisely, some background information about each person with dementia was collected at the beginning of the study. The IPS data, daily reports, and stress rating of each person with dementia were collected from weeks 2 to 8. Lastly, the interviews were conducted at the end of this study and transcribed verbatim. The setup of the location data collection in the ward is shown in Figure 3.

**Table 2:** Details of collected data in the study.

Category of data	Collection time	Description
Background information	At the beginning of the study	Age, gender, basic clinical background, and typical behaviors of people with dementia
Indoor positioning system data	Continuous daytime collection from weeks 2 to 8	Location of people with dementia and caregivers
Daily reports	Once or a few times per day from weeks 2 to 8	Perception about when and where did the person with dementia get stressed, what did he/she do, and what did the caregiver do to reduce his/her stress
Stress rating	Every half an hour during the waking time of the residents from weeks 2 to 8	Perception of caregivers about the level of stress expressed by the person with dementia (color code used from low to high stress: green, yellow, orange, and red)
Feedback on the digital platform	At the end of the study	Semistructured interview on discovered insights and corresponding actions (if any), usefulness, and desired improvements of the digital platform

### Data Analysis

#### Overview

Three analyses were performed to fulfill the research goals outlined above. The first analysis examined the availability and utility of the data collected by the digital platform. The second analysis investigated what types of insights and actions, if any, could be generated by the care team via using the digital platform. The third analysis studied the perceived usefulness and desired improvements regarding the digital platform.

Concerning the transferability of this study, we followed the strategies by Polit and Beck [36], which recommend that a sufficiently detailed description of the context is needed for the study results to be meaningful to other researchers. Transferability is defined as “the degree to which the results of qualitative research can be transferred to other contexts or settings with other respondents” [37]. Describing not only the study results but also their context could help the study results become meaningful to an outsider [37]. Hence, basic information about the nursing home where the research was conducted and a description of the three people with dementia are provided in Multimedia Appendix 1 and Multimedia Appendix 3, respectively.

#### Analysis 1: Data Availability and Utility

The aim of this analysis was to determine whether the digital platform could collect adequate data for the intended purpose of identifying behavioral patterns. With “adequate,” we implied the amount of data that could enable behavioral patterns to be revealed via visual inspection. Visual inspection, sometimes referred to as visual analysis, is the most widely used and recommended method for interpreting single-subject data [38,39]. This method involves the researcher analyzing the data visually, which allows for a holistic evaluation for understanding the idiosyncrasies present in the data for each subject [40]. The amount of data collected depends both on the technical functioning of the IPS and the participants’ adherence (ie, keeping the tags charged and connected, and wearing the tags correctly).

**Figure 4:**
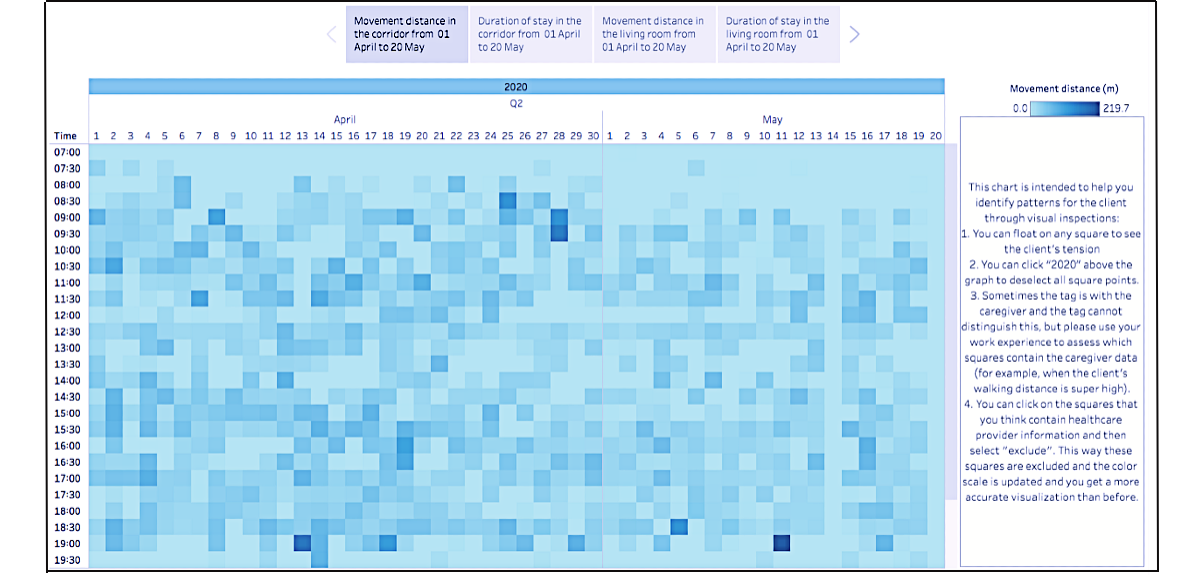
Movement distance in the corridor for participant 1 from April 1 to May 20, 2020, for each day in the daytime (tile plot).

**Figure 5:**
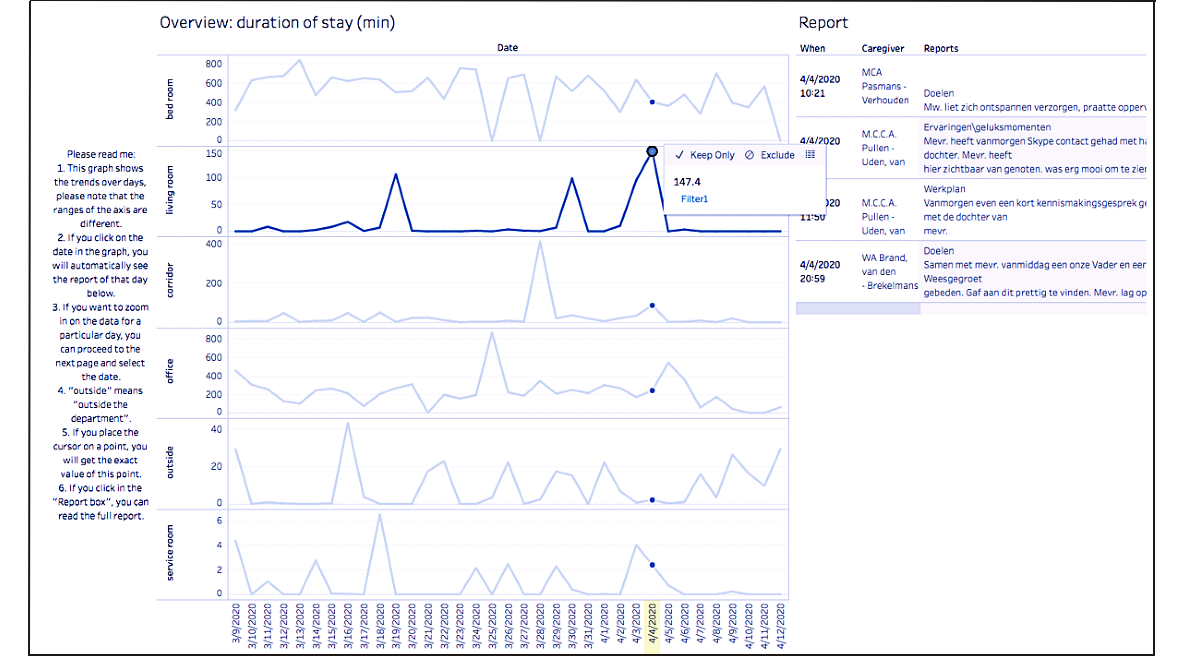
Duration of stay for participant 2 in all possible rooms in the ward per day in the daytime and corresponding daily report (combined plot).

**Figure 6:**
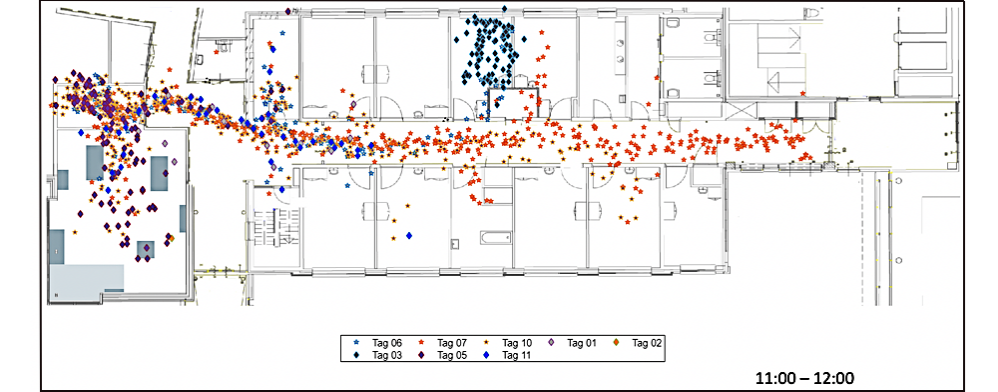
Movement trajectories of people with dementia (tags 1-3) and caregivers (tags 4-11) in the ward from 11:00 AM to 12:00 PM on April 16, 2020 (mapping plot).

The researchers examined the data availability by checking the proportion of the study period for which data were available for each person with dementia. Visual inspection was then applied to identify whether behavioral patterns could be revealed for each person with dementia. If behavioral patterns were found, these findings would be validated with the care team. Three types of visualizations were created in the end, namely, tile plots (example in Figure 4), combined plots (example in Figure 5), and mapping plots (example in Figure 6). Only tile plots were used for the purpose of visual inspection (the guide for visual inspection and the validation process with the care team can be found in Multimedia Appendix 4).

#### Analysis 2: Types of Insights and Actions

The aim of this analysis was to explore what types of insights and actions, if any, could be generated by the care team from using the digital platform. To prepare the caregivers for the data examination, we sent them an email with a link to the three types of visualizations and instructions. The caregivers were asked to examine the data individually, and after that, they provided their insights at the care plan meetings. In this way, the insights were discussed within the care team. The caregivers were notified that they could contact the research team if any questions arose during the examination process. Being involved in developing the digital platform previously, the care team has gained experience in this type of data examination.

During the interview, each interviewee was asked if he/she had identified any insights from data examination and, if so, whether he/she would take any actions based on the insights. The interview guide can be found in Multimedia Appendix 5. Based on thematic analysis of the interview transcripts, GW and AA categorized the types of insights and actions into themes according to the six-step guidance by Braun and Clarke [41].

#### Analysis 3: Usefulness and Future Improvement

The aim of the third analysis was to evaluate the perceived usefulness of the digital platform by the care team. During the interview, each interviewee was asked if this digital platform was useful to his/her work and what could be improved. The interview guide can be found in Multimedia Appendix 5. Thematic analysis was conducted by GW and AA following the same guidelines as in Analysis 2.

We acknowledge that people with dementia were among the key stakeholders in this project. Since the people with dementia were in the later stages of dementia, we chose not to involve them in data examination and interviews. Instead, we involved care team members as they evaluated and monitored the current physical and emotional states of the residents daily and responded to their needs. All this information contributes to the personal care plan. For the people with dementia, their data were collected and studied, and the generated insights were shared with the care team and hence contributed to a timely update of their personal care plans. Given that dementia is progressive, receiving up-to-date care is essential for the well-being of people with dementia. In this way, people with dementia can benefit from participating in this project.

## Results

### Data Availability and Utility

The researchers analyzed whether location data collected over a longer period of time could reveal any behavioral patterns of people with dementia, such as trends and fluctuations in measured parameters within a day or over days. After the improvement of the tag design, the amount of missing data was reduced in comparison to the preceding study. However, there were still cases where no data were collected for a whole day. For example, two days of data were missing over the 7-week period for participant 1, as shown in Figure 4 (May 14 and May 20, 2020). Four days and three days of data were missing for participants 2 and 3, respectively. The missing data were mainly associated with the incorrect use of tags by the caregivers (ie, placing the tag on the wrong person or forgetting to charge or use the tags). Compared with a preceding study, the amount of missing data decreased [27]. Specifically, 3 days out of 7 weeks (on average) contained missing data in this study. In contrast, 7 days out of 15 days contained missing data in the preceding study [27].

It seems that the missed data did not inhibit the potential for identifying behavioral patterns via visual inspection. To illustrate, we present a tile plot of the location data for participant 1 from April 1 to May 20, 2020, in Figure 4. Specifically, this figure visualizes the movement distance of this person with dementia in the corridor each day in the daytime, where the color intensity corresponds to the movement distance (ie, light color: short distance; dark color: long distance). From Figure 4, participant 1 was found to have a longer movement distance in the corridor in April than in May and rarely moved in the corridor around noon (which corresponds to lunch time).

In general, this visualization in Figure 4 is representative of all three micro case studies in terms of the availability and utility of the location data (more tile plot examples can be found in Multimedia Appendix 6). The data collected over this period of time were adequate for behavioral patterns to be identified via visual inspection, despite some missing data. When discussing with the care team, we found that the daily patterns were usually within the expectations of the care team, mostly because they coincided with the daily routines of the participants (eg, the care team expected participant 1 to be in the living room at lunch time). In summary, the daily patterns uncovered about each person with dementia validated the experience and knowledge of the care team. Yet, the trends and periodic patterns over the days were usually new insights to the care team (eg, the care team did not know the total movement distance of participant 1 decreased over time).

In addition to tile plots, two more types of visualizations were also developed. The combined plot presented the daily reports and daily location data of the person with dementia side by side, as shown in Figure 5. Specifically, Figure 5 shows the duration of stay of participant 2 in all possible rooms in the ward each day, and clicking a data point on the graph provides the exact value of the duration of stay and the daily reports of this corresponding date. The main utility found for visualizing the duration of stay and daily reports together was to identify when the participants were under stress and what was done to reduce their stress. From previous research, we know that people with dementia might be sent back to their bedrooms if they are showing symptoms of stress, since there is a common belief within the care team that reducing the number of stimuli could help one to reduce stress, and the bedroom is regarded to have fewer stimuli than the rest of the ward. The daily reports normally recorded what happened and what was done around the stress moments.

The mapping plot presents the location data of all participants in their movement trajectories in the ward every hour in the daytime, as shown in Figure 6. For this visualization, on selecting a specific participant’s location trajectory, the trajectory of the selected person is highlighted, with all other participants’ trajectories visible in the background. The main utility found for this visualization was to identify if any person with dementia exhibits unique movement patterns spatially. From Figure 6, participant 3 was found to walk in circles when in the bedroom alone, which the care team indicated as insightful. The care team wondered if participant 3 was looking for a way out. From past experience, they know that participant 3 tends to walk when under stress, and this visualization made them realize that participant 3 walked in the room for a while after being sent to the room for relieving stress. This type of visualization was created since the care team had expressed that they would like to know the exact locations (eg, where in a room) and movement trajectories of the residents to uncover detailed movement patterns [27].

### Types of Insights and Actions

#### Types of Insights

The reported time spent on data examination ranged from 0.5 to 1.5 hours. Twenty-nine insights were generated by the caregivers, the doctor, the psychologist, and the dietitian based on their interpretation of the data visualizations. The manager did not find any insights. These insights were grouped into 13 subthemes that are under three themes. The first theme, client level (the care team refers to residents as their clients), includes (1) day structure according to care plan, (2) moments of unrest, (3) unusual movement trajectory, (4) behavior change over time, (5) physical activity over time, and (6) effect of medication. The second theme, ward level, includes (7) interaction with caregivers, (8) interaction with other people with dementia, and (9) dining environment. The third theme, team level, includes (10) more detailed reports, (11) tag usage, (12) behaviors of caregivers, and (13) workflow. These themes and subthemes of insights are presented in Table 3, with each theme illustrated by an example quote. A detailed analysis of all the interviews can be found in Multimedia Appendix 7.

**Table 3:** Types of insights generated from data examination (the care team refers to residents as their clients).

Theme and subtheme		Example quote^a^
**Client level**		
	Day structure according to care plan	“He has a day structure, in which he goes to the toilet two times a day around 11 am and 3 pm. From the data, sometimes he goes to the toilet once, and sometimes he does not go to the toilet at all. Because of his agitation, he forgets to ask to go to the bathroom, and then we might forget about it too.” [Caregiver 1] (mapping plot + stress rating)
	Moments of unrest	“He more often gets agitated in the afternoon than in the morning. I think maybe after lunch; he starts to think about the next step; he cannot wait to have something to eat. Sometimes he asks for food; sometimes, he asks what are we going to do next? He is a bit bored in the afternoon.” [Caregiver 1] (tile plot + daily report)
	Unusual movement trajectory	“I see his usual pattern from his room to the living room and to the kitchen. There has been one time to the back of our ward. Why? I don't know, but that's not the route he usually takes. Yeah.” [Caregiver 3] (mapping plot + daily report)
	Behavior change over time	“It seems that the connection (between restlessness and stress) is no longer there … perhaps his stress manifests itself less in movement and more in shouting. So, the restlessness has moved from motor to verbal. That is something I know from experience.” [Doctor] (tile plot + stress rating)
	Physical activity over time	“You can see that in the morning he is more active. And during the day his walking distance gets less.” [Caregiver 3] (combined plot)
	Effect of medication	“He has medication at 8 am, 12 pm, 5 pm. When he is restless, he gets extra antipsychotic medicine. I sometimes noticed the medicine is effective and sometimes not.” [Caregiver 1] (combined plot)
**Ward level**		
	Interaction with caregivers	“It strikes me that he is relatively alone when he goes back and forth (in the corridor). Except at 5-6 pm when there is care routine, and he is (with somebody and) keep moving back and forth (in the corridor).” [Doctor] (mapping plot)
	Interaction with other people with dementia	“Most of the time, I would like to see my clients only (in the digital platform), but sometimes when my clients interact a lot with other clients, then it's also sometimes useful to know what other clients are doing.” [Dietitian] (mapping plot)
	Dining environment	“I wonder what they have been doing during mealtime? Because some people I don't see in the living room (where the meal is served). It would also be nice if there is some quiet time around mealtime because when there's a lot of distraction, some people forget to eat or go walking.” [Dietitian] (mapping plot)
**Team level**		
	More detailed reports	“Sometimes even when a high stress level is recorded for the client, there is no corresponding daily report to explain what happened.” [Psychologist] (combined plot)
	Tag usage	“If I look at 3rd May, yeah, I think he had his tag put on only at 11:30. Sometimes, on 14th May, he doesn't have it (the tag) with him at all.” [Caregiver 3] (tile plot)
	Behaviors of caregivers	“I am also impressed by the distance traveled by the care staff … the staff is “more restless” than the residents. What's normal? I find it interesting to reflect on that with the team.” [Doctor] (mapping plot)
	Workflow	“It also gives me insight about all our daily things. And that's I think that my colleagues they are…that they are more interested to see this.” [Caregiver 3] (mapping plot + daily report)

^a^The main data sources on which the insights were based are presented in parentheses after each example quote.

#### Types of Actions

Except for the manager, the rest of the care team participants wanted to take some actions based on the insights they uncovered. Their intended actions were grouped into six subthemes that are under two themes. The first theme, investigation, includes (1) discuss causes behind insights, (2) monitor day structure, and (3) evaluate changes in care plan. The second theme, implementation, includes (4) change in care plan, (5) work practice, and (6) prediction. These themes and subthemes of actions are presented in Table 4, with each theme illustrated by an example quote. A detailed analysis of all the interviews can be found in Multimedia Appendix 7.

**Table 4:** Types of actions based on the insights generated.

Theme and subtheme			Example quote
**Investigation**			
	Discuss causes behind insights	“I see this client moves a lot when he is in high stress level around 3 pm; we don't know what is going on, maybe because he wants to go to the bathroom, or he has nothing to do. It is a signal that things are not OK for him; hopefully, we can find reasons for this” [Psychologist]	
	Monitor day structure	“This allows us to see what has or has not already been offered in a day. This is easy to look back.” [Caregiver 2]	
	Evaluate changes in care plan	“We decided…to let him go to bed earlier. And I hope when we see a new view (visualization) then I can see a difference in that (stress rating). To get to know if it's helpful for him.” [Caregiver 3]	
**Implementation**			
	Change in care plan	“We should bring him to the bathroom twice a day; this could help him to relax. I will discuss with my colleagues and update the care plan on this.” [Caregiver 1]	
	Work practice	“There should always be a caregiver in the living room when he is in the living room. He doesn't like to be alone.” [Caregiver 1]	
	Prediction	“From the data, I know when the client is more likely to get tense. Previously, I only observe their behaviors to get to know if he is tensed or not. It is predictive.” [Caregiver 3]	

### Usefulness and Future Improvement

#### Usefulness

The feedback of the care team participants on the usefulness of the digital platform for their work was categorized according to their professions. Their feedback is summarized in Table 5 with example quotes. A detailed analysis of all the interviews can be found in Multimedia Appendix 7.

**Table 5:** The perceived usefulness of the digital platform.

Profession	Perceived usefulness	Example quote
Caregiver (n=3)	Provide evidence for discussion and for confirming feelings	“It is nice that we use the data as the evidence when we discuss what we see with the doctor and psychologist.” [Caregiver 3] “The data are confirmations of our feelings towards the client.” [Caregiver 2] “I feel they are a confirmation about what I already knew.” [Caregiver 1]
Doctor (n=1)	The insights are useful, but more scientific evidence is needed	“I think it is getting better and better. The clinical relevance is still complicated; for each client, we can do something with that, but for a scientific basis, not only a feasibility study but also a real clinical study is needed.”
Psychologist (n=1)	Triangulate subjective report of the caregivers with collected data	“For me, since I am not in the ward myself, I normally talk with the caregivers; it is good to see how often he is in stress (from the visualizations).”
Dietitian (n=1)	Need more data related to food and dining	“It tells me where the person is, how long the person stays there. So, it gives some data for me. Yes. And stress. But it doesn't mean a lot for the dietitian. It doesn't say a lot about food; it is more about what's being done.”
Manager (n=1)	Digital platform is not helpful for my work	“Is it helpful for my work? not so much, because if I must put a conclusion, I have to have more data, more in an overview … I couldn't draw any conclusions from this data.”

#### Future Improvement

The care team participants formulated 23 key areas for developing the digital platform further. These were grouped into 10 subthemes that are under three themes. The first theme, data collection, includes (1) more types of data, (2) define whose data to collect, and (3) reliability of stress rating. The second theme, data visualization, includes (4) personalized parameters, (5) filter data by time, and (6) less clicking. Finally, the third theme, data examination, includes (7) reduce the examination time, (8) who should examine the data, (9) develop an examination workflow, and (10) automatic notifications. These themes and subthemes of areas of improvement are presented in Table 6, with each theme illustrated by an example quote. A detailed analysis of all the interviews can be found in Multimedia Appendix 7.

**Table 6:** Areas of improvement for the digital platform.

Theme and subtheme			Example quote
**Data collection**			
	More types of data	“What I missed is the actions, the interventions that the team members have done and what the effect is on the behavior of the clients; for me, it's hard to find any conclusion about examining this data.” [Manager]	
	Define whose data to collect	“We have known him for a long time … so we have done lots of analysis of his behaviors. I think these data will give much more information if it is someone new, who we don't know much about.” [Psychologist]	
	Reliability of stress rating	“Sometimes, stress-rating does not match the daily reports … that is very unfortunate because you cannot see many things properly … I don't know if there is any more convenient method for this (stress-rating).” [Doctor]	
**Data visualization**			
	Personalized parameters	“We only know the distance and how long he has been in the corridor. We would like to know how many times he moved back and forth in the corridor; this indicates his agitation.” [Caregiver 1]	
	Filter data by time	“It would be helpful to see what the person is doing at a time they should eat … that would tell me where the person is at mealtime or is he walking around? And that’s interesting. If I can select the time, then that would be nice.” [Dietitian]	
	Less clicking	“If I can see the report of the day when I hover on the data of that day, that would be good, I don't have to select the date for the report, and it will make the process faster.” [Psychologist]	
**Data examination**			
	Reduce the examination time	“This is new to me, I enjoyed doing it once. However, it is very difficult to look at the data when I have to care for clients.” [Caregiver 2]	
	Who should examine the data	“I noticed that caregivers have a lot of trouble in analyzing the data. And I think that’s … it has several reasons. But one of the most, I think the most important is that they are not used to analyzing data.” [Manager]	
	Develop an examination workflow	“We just talk about these in our regular meetings. It could be good if someone would first look at the data so that the data is not new for everybody … and discuss with the team in the regular meetings. During the meeting, everyone can discuss if they see the same things and why or why not.” [Psychologist]	
	Automatic notifications	“It would be nice if the device can generate some insights automatically to help us with the examination over time; for example, it can tell us when the data deviates from the baseline.” [Caregiver 1]	

## Discussion

### Principal Results

By evaluating the digital platform in a real-life context with potential users for 7 weeks, this study gained insights on applying data-enabled design for personalizing dementia care in a nursing home. The results demonstrated the potential for the data visualizations in the digital platform to reveal behavioral patterns despite the missing data. In addition, we identified three main types of insights generated from data analysis, two main types of corresponding actions, the perceived usefulness of the digital platform, and three areas for its improvement.

#### Implications of Analysis 1

The results showed that integrating the digital platform into the nursing home environment might offer an opportunity for the care team to uncover the behavior patterns of the residents and personalize care plans accordingly.

A study on adapting mobile and wearable technology to support and monitor rehabilitation for people with dementia in the home environment has discussed that the monitoring approach could replace traditional methods for behavior analysis (eg, questionnaires) with four advantages [42]. The findings from this study coincide with three of the four advantages identified, which are as follows: the behavioral patterns identified by monitoring could be of higher resolution, data quality is independent of human recall, and collaborative care could be facilitated by sharing the data visualizations. The last advantage identified by this previous study is that the workload of the care team could be lowered by the monitoring approach [42]. In our case, the interplay of monitoring with assistive technology and the current practice of the care team was explored; hence, the time spent by the care team in managing the tags and examining the data was factored in. In this way, the overall workload increased in the short term, which was the main pain point mentioned by the care team.

The workload of the care team members might decrease in the long term as they gain experience with managing the tags and examining the visualizations. Meanwhile, the future development of the digital platform should focus on autonomizing some tasks for the care team. Moreover, BPSD management contributes to a high percentage of caring workload [43], and the insights generated from using the digital platform may offer novel and personalized ways for managing BPSD. Thus, using the digital platform would potentially enable BPSD management to be easier for the care team. In this study, most members of the care team identified the potential added value of this digital platform to their care practice; besides, they became more familiar with data collection and examination. Higher familiarity and having perceived needs have been recognized as two contributing factors for better user adherence [42]. Hence, a longer-term evaluation is needed to examine if the effort and time invested in using the digital platform could be outweighed by the effort and time saved from using it.

#### Implications of Analysis 2

The three main types of insights generated after data analysis were at the client level, ward level, and team level. Even though the initial aim of introducing the digital platform was to collect data that are unique to each individual, the care team generated insights that were not limited to the clients. This is because the context in which a person with dementia lives is also an important part to consider when personalizing dementia care, which reveals the importance of context in personalized dementia care. We recommend that future researchers and developers pay attention to this.

**Figure 7:**
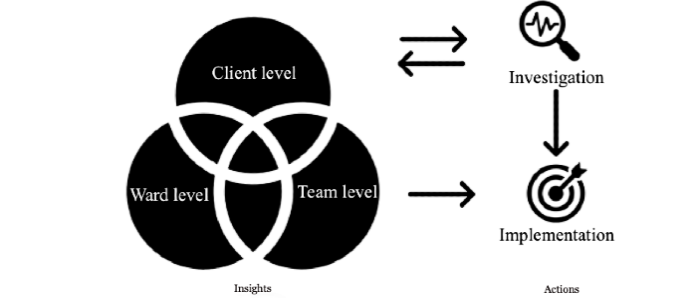
Tentative relations between the types of insights and types of actions generated in this study.

Although the types of insights and the types of actions were categorized in the Results section, they were interrelated in various ways. A tentative overview of the interrelations among them is shown in Figure 7. From the “Insight” section of this figure, some insights at the client level, ward level, and team level mutually influence one another. For example, when some caregivers increase awareness about their behaviors (team level), their interaction approach with the residents might change (ward level), and this may lead to a reduction in the frequency of BPSD moments for the residents (client level). Moreover, due to the interrelations between these insights, uncovering one insight may lead to the discovery of other insights. For example, realizing that the day structure of a resident is usually disrupted at certain times (client level) may trigger the care team to recall if there are any differences in this resident’s environment at these times (ward level) or to reflect if there are any difficulties in the workflow of the caregivers around these times (team level). Despite these overlaps, we feel that all the themes and subthemes provide a set of specific types and subtypes of insights, respectively. Discussing the various separated themes and subthemes naturally results in making sense of their interconnections.

Zooming out from the “types of insights” in Figure 7, we postulate that these insights interact with the two types of actions identified, which are “investigation” and “implementation.” When an insight is in the form of a statement (eg, the client forgets to go to the bathroom when agitated), the care team will implement it in care practice (eg, bring the client to the bathroom even though he did not ask for it); however, when an insight is in the form of a question (eg, what has the client been doing during dinner time?), the care team will inspect to find the answer by “investigation” (eg, meeting, observation, and collect and examine more data), and this process will lead to more insights. When the care team members are certain about their findings after more investigation, they will then implement them in care practice.

These types of insights and actions together with their interactions could also be applied in evaluating assistive technologies in other contexts of personalizing dementia care in nursing homes. We hence encourage future researchers and developers to use Figure 7 as a conversation guide when evaluating these assistive technologies with a care team and be open-minded about more types of insights and actions generated.

#### Implications of Analysis 3

The people with dementia, caregivers, doctor, psychologist, dietitian, and manager were among the key stakeholders for the digital platform. Previous studies have found that caregivers’ acceptance of assistive technologies is vital for successful integration and usage of these technologies in nursing homes [24]. We would like to add that, in the case of managing BPSD with assistive technologies, all members of the care team should be involved. This is because a group effort is needed in incorporating insights from the data examination into the current care practice for BPSD management. Different stakeholders tend to have different interests in a project. In our previous study, we identified that the main value held by the care team is to provide better care to people with dementia. As the digital platform was implemented in the ward for a longer period of time, we noticed that, despite the main value, it is important to cater for the different interests among stakeholders. Specifically, most members of the care team identified the potential added value of this digital platform in their care practice. The manager was mainly interested in the efficiency of care in the ward and therefore saw fewer benefits from the digital platform for her work.

To manage the competing needs stemming from different stakeholders, we recommend future studies to apply a multicriteria analysis to prioritize the stakeholders involved [44]. For example, the manager might be interested in different aspects of care in comparison to other members of the care team and thus could be involved differently. Knowing the reasons behind the low acceptance of some stakeholders and prioritizing stakeholders early on could help with the development of future assistive technologies for dementia care in nursing homes.

**Figure 8:**
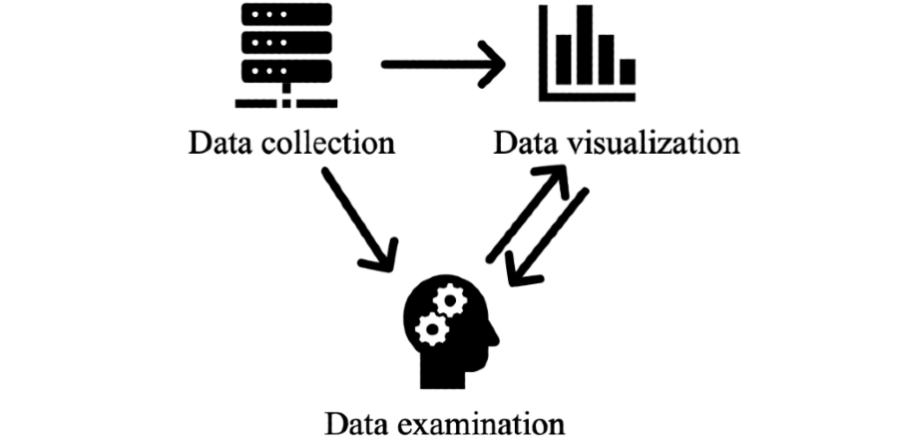
Tentative connections of the three improvement areas identified for the digital platform.

We postulate that the three main areas of improvement identified for the digital platform, namely, data collection, data visualization, and data examination, are interconnected to each other. Their tentative connections are shown in Figure 8. First, a change in data collection would affect both data visualization and data examination. For example, introducing data about nonverbal behavior might lead to a change in previous data visualizations to accommodate this new data type, and the examination process may have to be adapted accordingly. Second, an update in data visualization tends to affect data examination more than data collection. For instance, incorporating the hovering option for quick report reading can reduce the data examination time directly, but data collection will not be affected. Third, the data examination process, in turn, tends to affect data visualization more than data collection. Take the inclusion of automatic notifications as an example; while data collection could be the same, additional visualizations might be needed for presenting the data based on which the algorithmic analysis is made. This is to ensure the transparency of algorithms to the users, which has been found to be of paramount importance in decision-making [45]. The researchers and developers are advised to be aware of the mutual influences of these three areas when developing similar kinds of assistive technologies.

### Reflection on Data-Enabled Design

Through the evaluation of the digital platform, we also learned about the value of data-enabled design in personalizing BPSD management. First, we experimented with the frequency and duration of collecting different types of data to explore the qualities and limitations of data as design material, as suggested by Bogers and van Kollenburg (who coined data-enabled design) [26]. In this way, the visualizations were perceived as relevant and meaningful to most members of the care team (eg, by increasing the frequency of stress rating). Second, after data had been collected over a longer period of time, the care team was able to gain insights from data visualizations that they otherwise would not have gained via traditional methods (eg, observation). The same has been found in previous data-enabled design studies [46]. Moreover, the care team generated insights at not only the client level but also the ward and team levels. This corresponds to the intention of data-enabled design as follows: “gain detailed and nuanced contextual, behavioral, and experiential insights” [26].

In addition, we adapted data-enabled design in several aspects to fit it in the dementia care context. First, even though one of our key stakeholder groups, people with dementia, was not involved in the data examination and interview sessions owing to cognitive impairments, we paid attention to their nonverbal behaviors when introducing the tags to them. Residents who showed signs of dislike toward the tags were excluded from the study, although their family members had signed the consent forms. We then included “design the tags to be more dementia friendly” on the to-do list. We found that more relevant design directions could be generated by combining data-enabled design with close observations of people with dementia (a way of co-designing with people having dementia [47]). In contrast, a previous data-enabled design study on developing a smart baby bottle only involved the parents in the design research [48]. Similar to people with dementia, babies can communicate their needs nonverbally, and more insights might be generated if the designers also involve the babies during the design process in the future.

Second, the data-enabled design approach was initially developed for designers to gain rich insights for their design projects. As stated by Bogers and van Kollenburg, “the aim of this approach is to, together with end-users, unravel the relevance, potential, and pitfalls of data in a specific context to design concepts that resonate” [49]. In our study, we identified that the care team could also gain insights about people with dementia and the contexts with this approach to improve their care practice. Since care team members are experts in understanding their clients and the care context, by involving them in the data examination, they can see things that designers cannot see. The designers can then learn from the insights generated by the care team and apply these insights in the design process. We, therefore, recommend the care team to be more involved in the data-enabled design process not only because the care team can gain more insights to improve their work, but also because the designers can learn more from working with the care team.

Finally, if one would like to apply data-enabled design in the dementia care context, an evaluation of the designed product (in our case, assistive technology) in the longer term is important. Both Figures 7 and 8 indicate that there is an interplay between the technology introduced and the current BPSD care in the nursing home. There is a growing body of research on understanding how technology innovations change the dynamics between the people involved in health care and care practices, and these changes could, in turn, alter the role of the introduced technology in daily practice over time [50]. This study adds to this body of work by gaining an understanding of the drivers and barriers of integrating assistive technology via a data-enabled design approach in the institutional dementia care setting.

### Limitations and Future Work

The main limitation of this study was that all the interviews were conducted in a one-to-one format. This is because finding a time slot suitable for all the participants was difficult given the fast pace and high uncertainty of the working environment in the nursing home. A potential shortcoming of this format is the lack of discussions within the care team, which might have stimulated more ideas during the interviews. The benefit of this format is that each participant can express his/her own views and interpretations without being influenced by other participants. The strengths of this study were that the digital platform was deployed in a real-life context and a wide range of stakeholders with different professional backgrounds was involved. Previous research has found that the use of different professionals’ perspectives is valuable in developing assistive technologies for people with dementia [51-53].

For future work, the digital platform could be improved based on the feedback from the care team. For instance, regarding reducing the data examination time, the manual process of data visualization is planned to be automated by algorithms. In addition, further algorithms could be developed for pattern analysis (to replace visual inspection) and for making predictions. The results of the algorithmic analysis could then be presented in the form of push notifications, with which relevant visualizations will be provided.

Moreover, it would be interesting to investigate if combining different types of visualizations could generate more insights. Although all three types of visualizations were sent to the care team at the same time, all participants discussed the visuals one by one, which indicates that combining visualizations might not have been explored during the data examination process. It could be that there was no explicit instruction for exploring visuals in a combined way, or all participants did not see added value when combining these visuals; therefore, these explorations were not mentioned in the interviews.

In this study, we presented an in-depth investigation with a care team that was insightful in understanding the value of the digital platform and the application of data-enabled design in developing assistive technologies for personalizing BPSD care. We, therefore, suggest researchers and developers consider this case study approach in the development of future assistive technologies for personalizing dementia care.

### Implication for Dementia Care During a Pandemic

This study was carried out during the lockdown period in the Netherlands because of the COVID-19 pandemic. At the beginning of the project, the research team (GW, AA, and TvdC) went to the nursing home to introduce and discuss the study both at the management level and at the ward level. The principal researcher (GW) then spent a few months developing the digital platform with the care team. When the nursing home shut down because of COVID-19, the principal researcher stayed in touch with the care team via email, Skype, and Zoom. In this way, the research team collected all the data without going to the nursing home and conducted the interviews online with the care team. In hindsight, this digital platform has the potential to help care team members and researchers gain insights about each person with dementia remotely, which might be valuable in case of the current pandemic and future pandemics. In addition, the family members of people with dementia could benefit by using the digital platform to stay updated about the situation of their loved ones in the nursing homes remotely, which could decrease the already high communication workload of the care team during a pandemic.

### Conclusions

By evaluating the digital platform developed, this study gained insights on applying data-enabled design for personalizing dementia care, specifically in BPSD management. The collected data demonstrated potential use for pattern detection. The types of insights and actions generated from using the digital platform were identified and found to be interconnected. The perceived usefulness of the digital platform was found to vary across the care team, and we uncovered three aspects of improvement for the digital platform. These findings could guide future researchers and developers in investigating similar assistive technologies for personalizing dementia care in nursing homes and beyond.

## Appendix

Multimedia Appendix 1 Contextual information about the study field.

Multimedia Appendix 2 Ethics approval letters of Delft University of Technology and Zorggroep Elde Maasduinen.

Multimedia Appendix 3 Contextual information about the people with dementia who participated in the study.

Multimedia Appendix 4 Guide for visual inspection.

Multimedia Appendix 5 Interview guide.

Multimedia Appendix 6 Examples of visual inspection with tile plots.

Multimedia Appendix 7 Detailed description of thematic analysis results.

Multimedia Appendix 8 CONSORT-eHEALTH checklist (V 1.6.1).

## Abbreviations

BPSD: Behavioral and Psychological Symptoms of Dementia
IPS: Indoor Positioning System
