# Supplementary material for: A Digital Platform for Facilitating Personalized Dementia Care in Nursing Homes: Formative Evaluation Study
Source: JMIR Form Res. 2021 May 28;5(5):e25705. doi: 10.2196/25705 (PMC8196358; doi:10.2196/25705)
Supplement: Multimedia Appendix 1 [file formative_v5i5e25705_app1.docx]

**Multimedia Appendix 1.** Contextual information about the study field.

| ***Care culture in the nursing home*** | ***Ward ambiance*** | ***Working structure of the care team*** |
| --- | --- | --- |
| Zorggroep Elde Maasduinen has multiple sites with 2,000 employees and 1,400 volunteers in total and cares for around 3,500 elderly people. In managing BPSD, the nursing home adopts the Dimentie-model [1]. The nursing home uses a standardized signal plan for the residents based on the Crisis Development model [2]. This model divides the stress level of a person into green, yellow, orange, and red, indicating no stress (green) to high stress (red). The effective measures carried out to help the resident relax at different stress levels are recorded and categorized on the signal plan. Based on the signal plan, the behaviors of the residents act as signals for their stress levels, and the caregivers can then react by adjusting the interaction style and environment for each resident according to the signal plan to reduce the stress for each resident. | In the ward where the IPS is installed, there are ten bedrooms available, and each resident has his or her own bedroom, which they can decorate with their personal belongings. The bedrooms open onto the main hallway, to which the living room is also connected. The living room is composed of an open kitchen and a dining room. The dining room is shared by these ten residents, and the decoration presents a home-like nostalgic atmosphere. This is where daily activities take place, such as watching television, chatting, singing, reading, or playing games. Most of the time, caregivers facilitate the residents with these activities in the living room. Three bathrooms are located on the two sides of the hallway. | There are four caregivers in the morning, three in the afternoon, and one in the night shift. Each day, depending on the state of the residents, the caregivers write one or more or no daily report(s) for each resident. In this study, their extra tasks are wearing the tags, help residents wearing the tags, and record a stress rating for the residents following the Crisis Development model. Each resident has a responsible caregiver, and all the residents share the responsible doctor, psychologist, and dietitian. The manager is mainly in charge of the performance of the ward and the wellbeing of the employees. The responsible caregiver is accountable for updating the care plans of the resident. Caregivers have a weekly meeting with the doctor and the psychologist and occasional meetings with the dietitian when the dietary plan needs to be changed. |

[1] “Methodology - D-ZEP Oleander.” https://dzep-oleander.nl/methodiek/ (accessed Mar. 30, 2021).

[2] R. Geelen, “Agressie en ontregeld gedrag: het Crisis Ontwikkelings Model (COM),” in *Dementiezorg in de praktijk - deel 2*, Bohn Stafleu van Loghum, 2015, pp. 167–184.
