# Supplementary material for: A Digital Platform for Facilitating Personalized Dementia Care in Nursing Homes: Formative Evaluation Study
Source: JMIR Form Res. 2021 May 28;5(5):e25705. doi: 10.2196/25705 (PMC8196358; doi:10.2196/25705)
Supplement: Multimedia Appendix 2 [file formative_v5i5e25705_app2.pdf]

zorggroep
elde
maasduinen

Parkweg 12
5282 SM Boxtel

Postbus 74
5280 AB Boxtel

Innovatie & Onderzoek
0411 - 634165
info@zge.nl

Boxtel, 2 juni, 2020

To whom it may concern,

I am pleased to declare that the board of directors and supervisors of Zorggroep Elde Maasduinen approved (on the 18th of November 2018) Ms. Gubing Wang and her promotors, from Delft University of Technology, for her research project Indoor Positioning for people with behavioral problems and dementia. The research has taken place on a specialized ward for people with severe behavioral problems and dementia (De Oleander) within one of our nursing homes.

I have met Ms. Wang and her promotors as dedicated researchers with knowledge about a specific area of research, namely sensors which generate specific data and insights we can use in taking care of people with dementia. Together we collaborated the past 2 years on gaining insights, so collected data can be used in care processes.

I am very sure that this project will succeed under supervision of Ms. Wang and her promotors.

In case you need any more information please find my contact details in above letterhead.

Best regards,

J.C.F. (Jef) Mol MSc., MHA
Director Innovation, Research & Temporary Care

Date 03-12-2018
Contact person Ir. J.B.J. Groot Kormelink, secretary HREC
Telephone +31 152783260
E-mail j.b.j.grootkormelink@tudelft.nl

Human Research Ethics Committee
TU Delft
(http://hrec.tudelft.nl/)

Visiting address
Jaffalaan 5 (building 31)
2628 BX Delft

Postal address
P.O. Box 5015 2600 GA Delft
The Netherlands

Ethics Approval Application: Managing Behavioural and Psychological Symptoms of Dementia (BPSD) through Design – a field study with indoor positioning system
Applicant: Wang, Gubing

Dear Gubing Wang,

It is a pleasure to inform you that your application mentioned above has been approved.

The application is approved under the condition that all data will be fully anonymized in publications and during presentations.

Good luck with your research!

Sincerely,

Prof. Dr. Sabine Roeser
Chair Human Research Ethics Committee TU Delft

Prof.dr. Sabine Roeser
TU Delft
Head of the Ethics and Philosophy of Technology Section
Department of Values, Technology, and Innovation
Faculty of Technology, Policy and Management
Jaffalaan 5
2628 BX Delft
The Netherlands
+31 (0) 15 2788779
S.Roeser@tudelft.nl
