# Supplementary material for: A Digital Platform for Facilitating Personalized Dementia Care in Nursing Homes: Formative Evaluation Study
Source: JMIR Form Res. 2021 May 28;5(5):e25705. doi: 10.2196/25705 (PMC8196358; doi:10.2196/25705)
Supplement: Multimedia Appendix 3 [file formative_v5i5e25705_app3.docx]

**Multimedia Appendix 3.** Contextual information about the people with dementia who participated in the study.

|  | ***Age*** | ***Gender*** | ***Basic information about the clinical background and typical behaviors*** |
| --- | --- | --- | --- |
| Participant 1 | 77 | Male | I have vascular dementia (MMSE=6). I like to be addressed by my first name. I don’t hear and see very well and am immobile. Please approach me from my right side, and do not touch me unexpectedly, because then I can be very shocked. Sometimes I can shout loudly and drive my wheelchair to the living room if I want to make something known. Sometimes I want to have everything organized in my way. I like watching TV, and I like to hold someone’s hand sometimes. I love having fun from time to time. A fixed day structure is very important to me so that things are recognizable and clear. |
| Participant 2 | 85 | Female | I have Lewy body dementia (MMSE=4), which sometimes gives me hallucinations. I could scream, hit objects, scratch others, speak in a monotonous tone if it happens to me. At other times I really like activities, such as “moving with music”. I like to go to church or the chapel (quiet room). I love to play games, especially Rummikub. I really enjoy chatting. A fixed day structure is very important to me, and sometimes I get nervous when I see strangers or people I do not like (the list of people I do not like can change from time to time). I spend most of my time in the wheelchair; I do not drive it myself, and people help me to move around. |
| Participant 3 | 80 | Male | I have Alzheimer’s disease (MMSE=3). Recently I had a bladder infection, which gave me discomfort. I have always been a wall painter, and I often carry out my “work” in the ward. I tend to get stressed after lunch, at which moment I walk a lot at high speed, clap my hands, and come too close to others. Letting me stay in my own room could calm me down. I really like puppies and dolls, and I can play with them for a long time. I also enjoy talking to others and sitting together with someone on the bench. Please approach me in a relaxed manner and with few words. Both my wife and my sons are important to me. |

MMSE: Mini-Mental State Examination score

The clinical background and typical behaviors of each PwD participant are extracted from the translated care plans (originally in Dutch), where the information is formatted in a first-person tone.
