# Supplementary material for: A Digital Platform for Facilitating Personalized Dementia Care in Nursing Homes: Formative Evaluation Study
Source: JMIR Form Res. 2021 May 28;5(5):e25705. doi: 10.2196/25705 (PMC8196358; doi:10.2196/25705)
Supplement: Multimedia Appendix 4 [file formative_v5i5e25705_app4.docx]

**Multimedia Appendix 4.** Guide for visual inspection.

**Guide for visual inspection**

This guide is inspired by the work by Parsonson and Baer [1], in where they discussed how to analyse line charts and bar charts for single-subject research in terms of baselines, trends, cyclic or recurring patterns. We have adapted the methods they used to suit the tile plots. Specifically, in our tile plot, the location of each tile indicates a particular time zone, a particular day, and the colour intensity of the tile indicates the value of collected data. Take Figure 1 as an example; the colour intensity shows the movement distance (m) for a client. We use the term “movement distance” to indicate that the clients can walk by themselves or move in a wheelchair. A darker colour indicates a longer movement distance; a lighter colour indicates a shorter movement distance. The duration of time for each tile is half an hour.


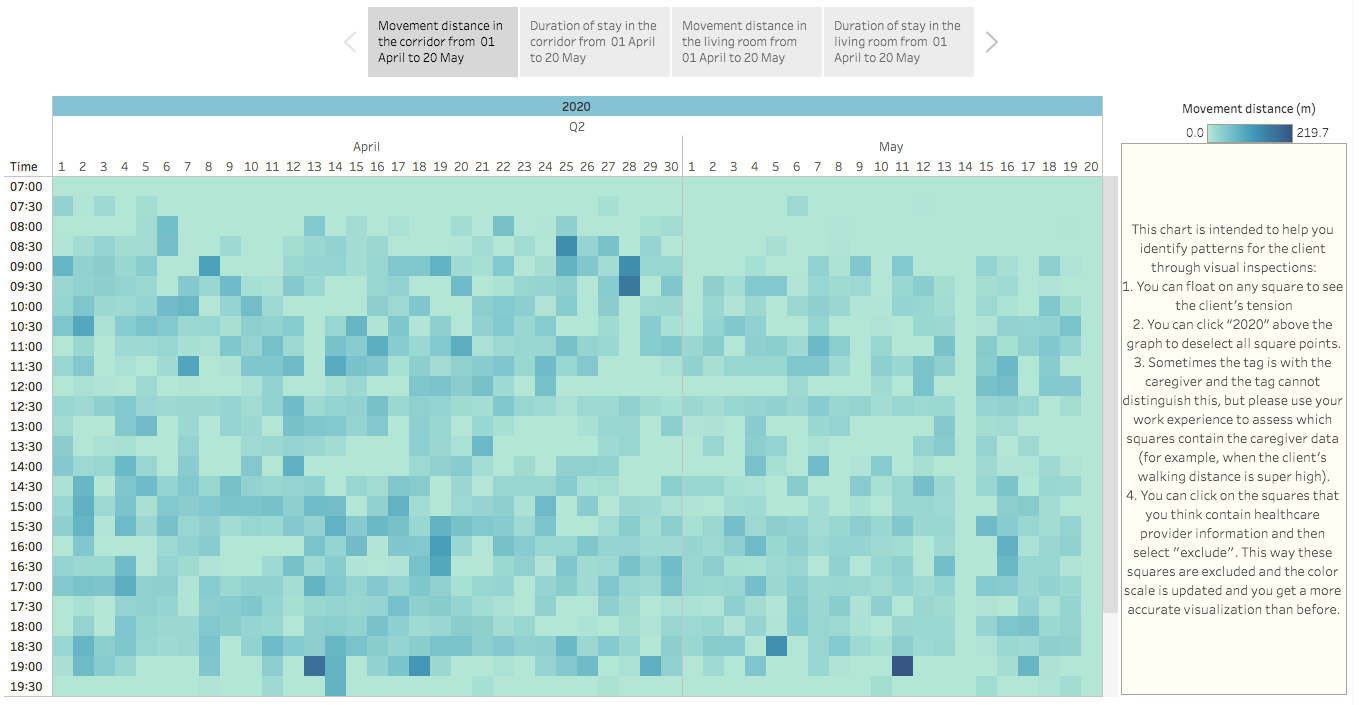


Figure 1. Movement distance in the corridor for Participant 1 from 1st April to 20th May each day during the daytime.

With the above information, here are the guides for visual inspection. It is required to have at least two visual inspectors. Each visual inspector examines the data individually and comes up with insights about trends and patterns; then, all the visual inspectors discuss their findings together until consensus is reached.

Step 1: Remove misuse data

- Remove misuse data according to the instructions on the digital platform (as shown in Figure 1, the instruction is in the text box besides the visualisation)

Step 2: Trend analysis

- From left to right (along the x-axis), is there a general change in the colour intensity of the tiles?
- From top to bottom (along the y-axis), is there a general change in the colour intensity across the tiles?

Step 3: Pattern analysis

- From left to right (along the x-axis), is there an alternating change in the colour intensity of the tiles? Where are the darker tiles, and where are the lighter tiles?
- From left to right (along the x-axis), are there any columns of tiles with similar colour intensity? Where are they?
- From top to bottom (along the y-axis), is there an alternating change in the colour intensity across the tiles? Where are the darker tiles, and where are the lighter tiles?
- From top to bottom (along the y-axis), are there any rows of tiles with similar colour intensity? Where are they?

Step 4: Validation with the care team

- After a consensus is reached among visual inspectors, invite at least one care team member to go through the visual inspection process (Step 1 to 3)
- Discuss with the care team member(s) about all the findings (from the visual inspectors’ side and from the care team’s side) until a consensus is reached

Step 5: Judgement of data adequacy

- After a consensus is reached between visual inspectors and the care team member(s), if there is no trend or pattern identified, then the data collected will be considered as “not adequate”; otherwise, the data collected will be considered as “adequate”.

Reference

1. B. S. Parsonson and D. M. Baer, “The analysis and presentation of graphic data,” in *Single Subject Research*, T. R. Kratochwill, Ed. 1978, pp. 101–165.
