# Supplementary material for: A Digital Platform for Facilitating Personalized Dementia Care in Nursing Homes: Formative Evaluation Study
Source: JMIR Form Res. 2021 May 28;5(5):e25705. doi: 10.2196/25705 (PMC8196358; doi:10.2196/25705)
Supplement: Multimedia Appendix 5 [file formative_v5i5e25705_app5.docx]

**Multimedia Appendix 5.** Interview guide.

## In general:

Online 30-min interviews with three caregivers, one doctor, one psychologist, one manager, one dietitian;

## Research questions:

R1: Did the personal data collected and visualized in this work contribute to personalized dementia care?

R2: What could be improved in the future for incorporating IPS (or IoT in general) in personalizing dementia care?

## Interview guide

**Thank you very much for participating in this research. With this interview, we intended to evaluate if the visualizations could support you in the personalized management of BPSD.** With “personalized” we mean that the care for each client is tailored according to the needs, preferences, daily routines etc of the client. We hypothesize these personal insights could be obtained from analysing personal data of the client. In this case, the personal data are the location data and daily report. **Your input is very valuable for us to evaluate the use of indoor positioning system and to think of future improvements**.

Prepare the iPad, share my screen

Do you mind if I take an audio recording? This is just so that I don’t miss anything. Thanks.

First of all, do you have any questions regarding the data visualization? Anything that is not clear to you?

If yes:

Discuss the unclear issues. (R2)

If no:

Start the interview.

Ask the question: can you explain…? When the expression of the participant is not clear.

May you give an example? May you give another example? What do you expect to see?

Interview questions:

1. Did you find any insights from analysing the data? (R1)

If yes, proceed with next question

If no, why do you think that you couldn’t find any insights? (R2)

1. Did you discover any insights that you are not aware of? (R1)

If yes, proceed with next question

If no, why do you think that these insights are not new to you? (R2)

1. Based on these insights, will you structure your work differently next time when care for Mr. A? (R1). (would you like to see some changes in the care for Mr. A in the caregiver level?)

If yes, what would be the changes you would like to make (see)? (R1)

If no, why do you think it is not necessary to restructure your work (make any changes in care)? (R2)

1. Will you communicate your insights with other members of the care team? (R1)

If yes, with whom? when? in what way? and what will you communicate? (R1)

If no, why do you think it is not necessary to communicate your insights within the care team? (R2)

1. Based on these insights, are there any modifications in the care provided to Mr. A. on the care team level that you think could lead to better, more personalized care for Mr.A? (R1)

If yes, what are the modifications? (R1)

If no, why do you think no modification is needed? (R2)

Then to conclude, here are 2 final questions about the data visualisations:

How long did you spend to analyse the data?

1. What do you find about the data analysis of the visuals? (R2)
2. What do you think could be improved regarding the visualisations? (R2)

Thank you – those were all the questions I have for you. Do you have any questions? If anything, else occurs to you after today, please don’t hesitate to let me know. Thanks again!

Wait for the participant to end the call.
