# Supplementary material for: A Digital Platform for Facilitating Personalized Dementia Care in Nursing Homes: Formative Evaluation Study
Source: JMIR Form Res. 2021 May 28;5(5):e25705. doi: 10.2196/25705 (PMC8196358; doi:10.2196/25705)
Supplement: Multimedia Appendix 6 [file formative_v5i5e25705_app6.docx]

# **Multimedia Appendix 6.** Examples of visual inspection with tile plots.

From Figure 1, Participant 1 tends to stay in the corridor around 11:30 and 15:30 based on the visualization of his duration of stay in the corridor.


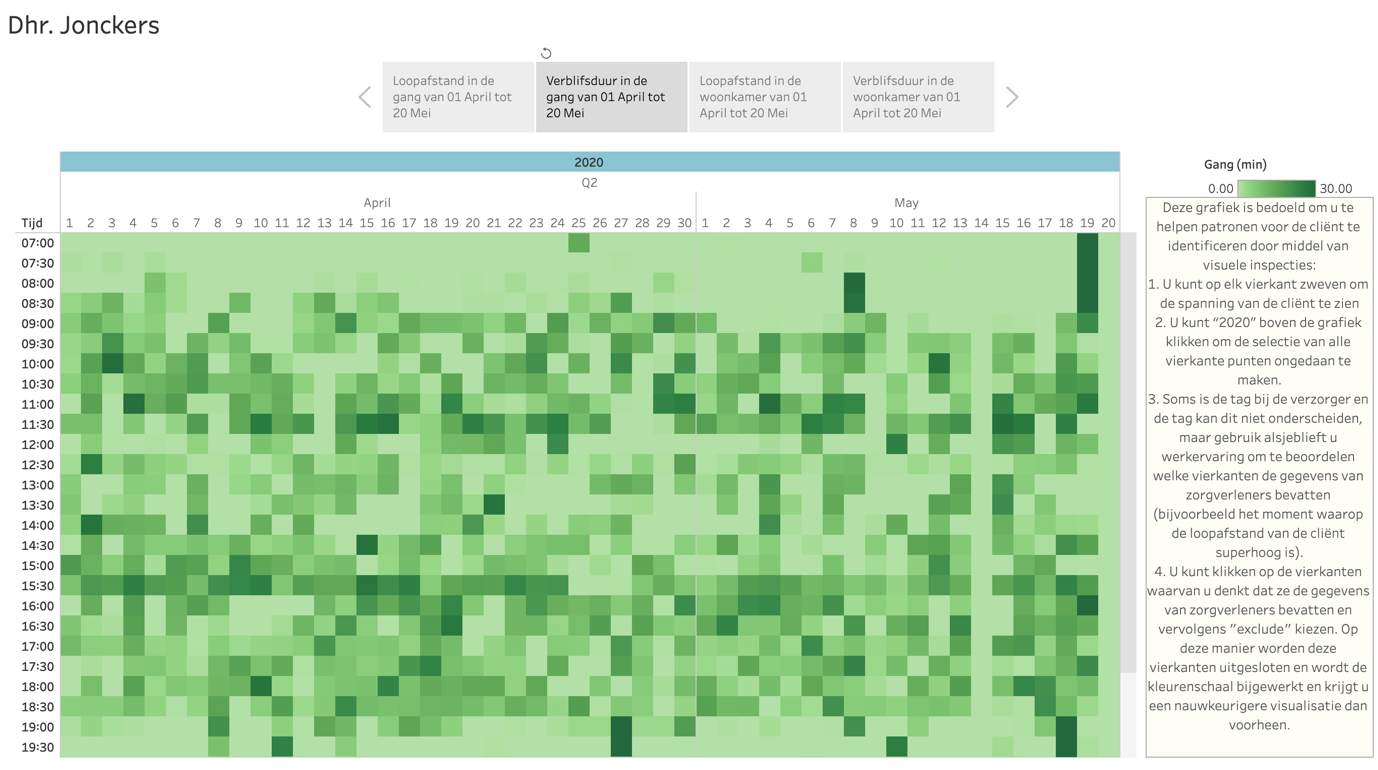


Figure 1. Duration of stay in the corridor for Participant 1 from 1^st^ April to 20^th^ May.

According to the visualization on the movement distance in the living room, as demonstrated in Figure 2, Participant 1 tends to move around in the living room around 12:30, and to the surprise of the researchers and care team, his movement distance from 13:00 to 15:00 oscillates almost every seven days.


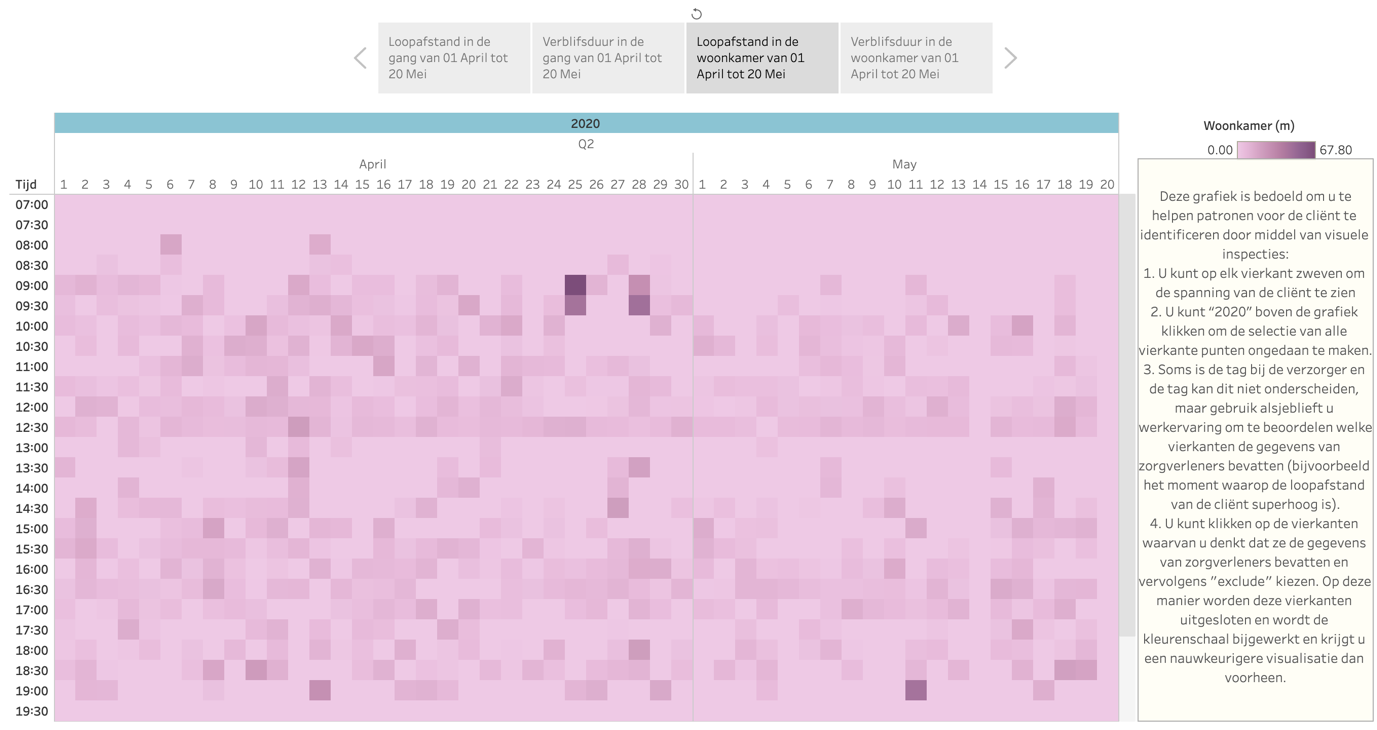


Figure 2. Movement distance in the living room for Participant 1 from 1^st^ April to 20^th^ May.

As for the duration of stay in the living room, as shown in Figure 3, Participant 1 tends to stay in the living room around 12:00 and 16:30 and spend less time in the living room around 12:30 than that of 12:00. His duration of stay in the living room from 13:00 to 15:00 also oscillates almost every seven days, which coincides with the oscillation in his movement distance in the living room. It has also been discovered that the time of entering the living room in May for Participant 1 is later than that of April, and he is usually not in the living room around 17:00 and leaves the living room around 19:00.


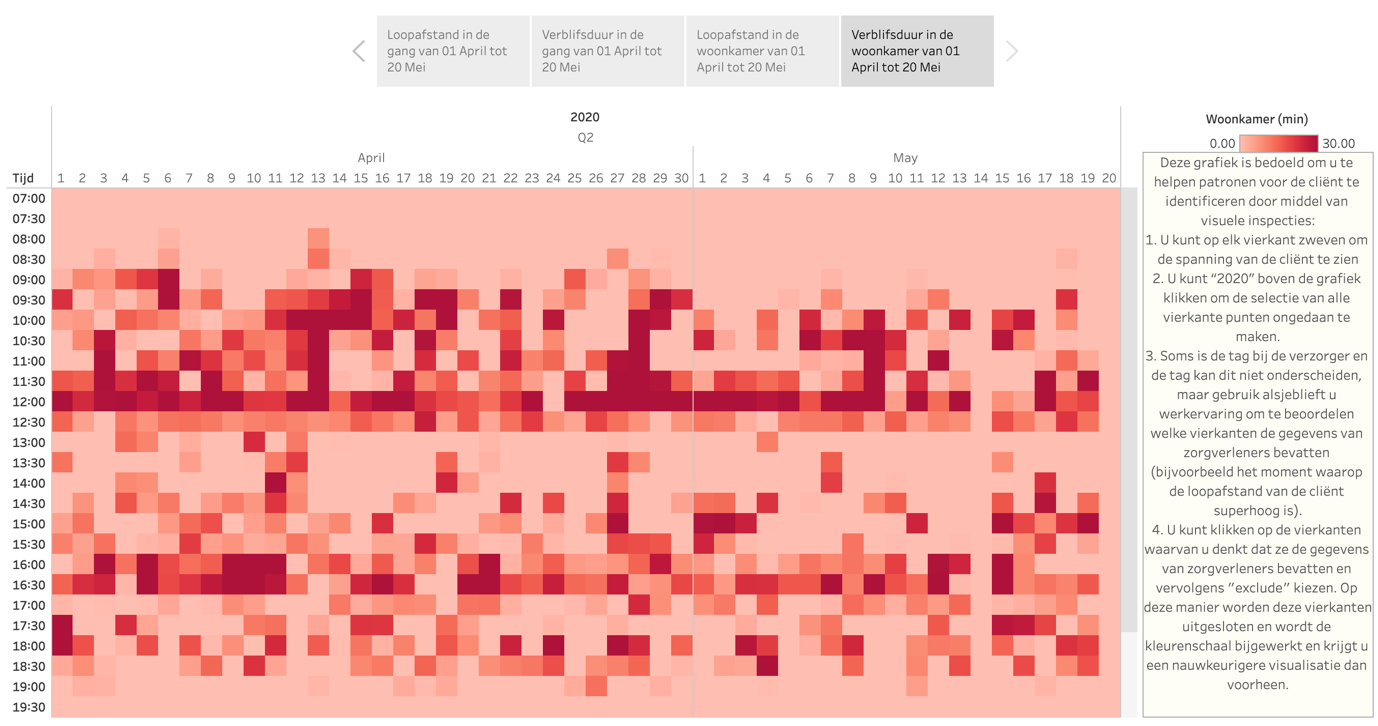


Figure 3. Duration of stay in the living room for Participant 1 from 1^st^ April to 20^th^ May.

Regarding the total movement distance, as demonstrated in Figure 4, Participant 1 moved around more in April than in May, and normally moved around about 12:30. Surprisingly, his movement distance from 12:00 to 15:00 oscillates almost evert three days over these seven weeks.


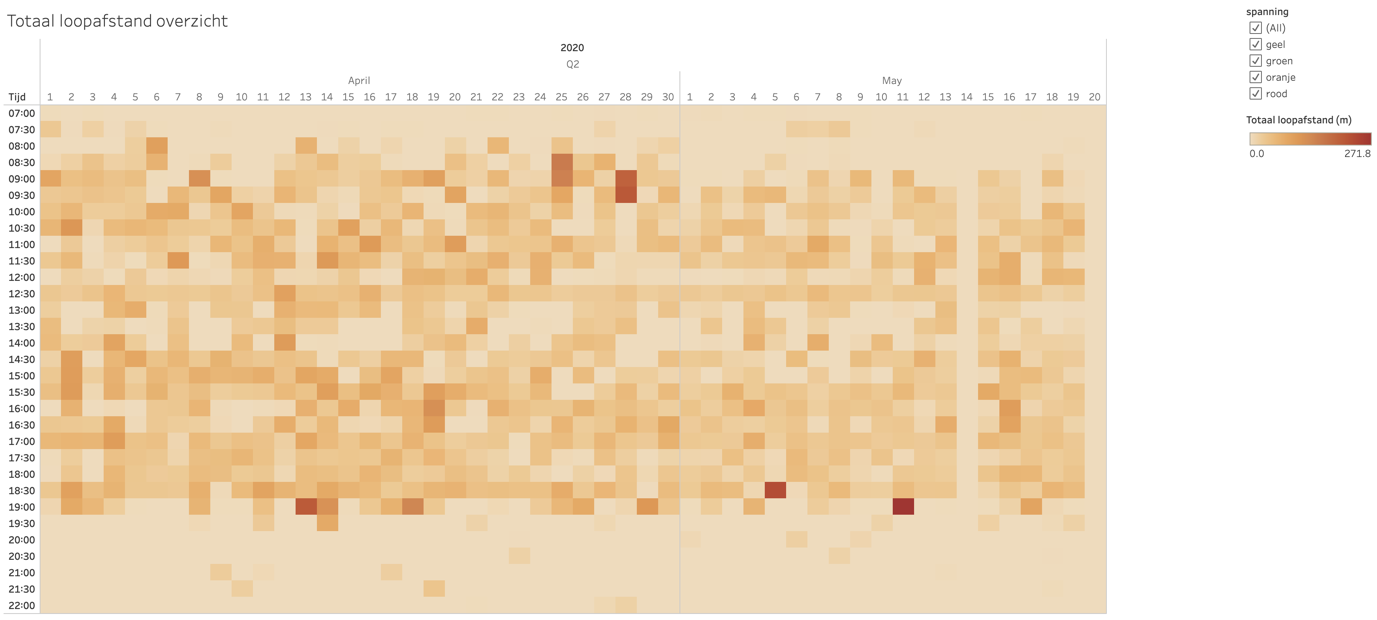


Figure 4. Total movement distance for Participant 1 from 1^st^ April to 20^th^ May.

The stress-rating filter was then added to all the previous visualizations discussed. As described in Table 2, the level of perceived stress of a PwD is categorized into four phases, which are, green, yellow, orange, red, from no stress to high stress. According to Figure 5, with only the “green” phase selected, the white spaces in this figure illustrate the times when Participant 1 was stressed. It has been found that Participant 1 got stressed more in the afternoon than in the morning.


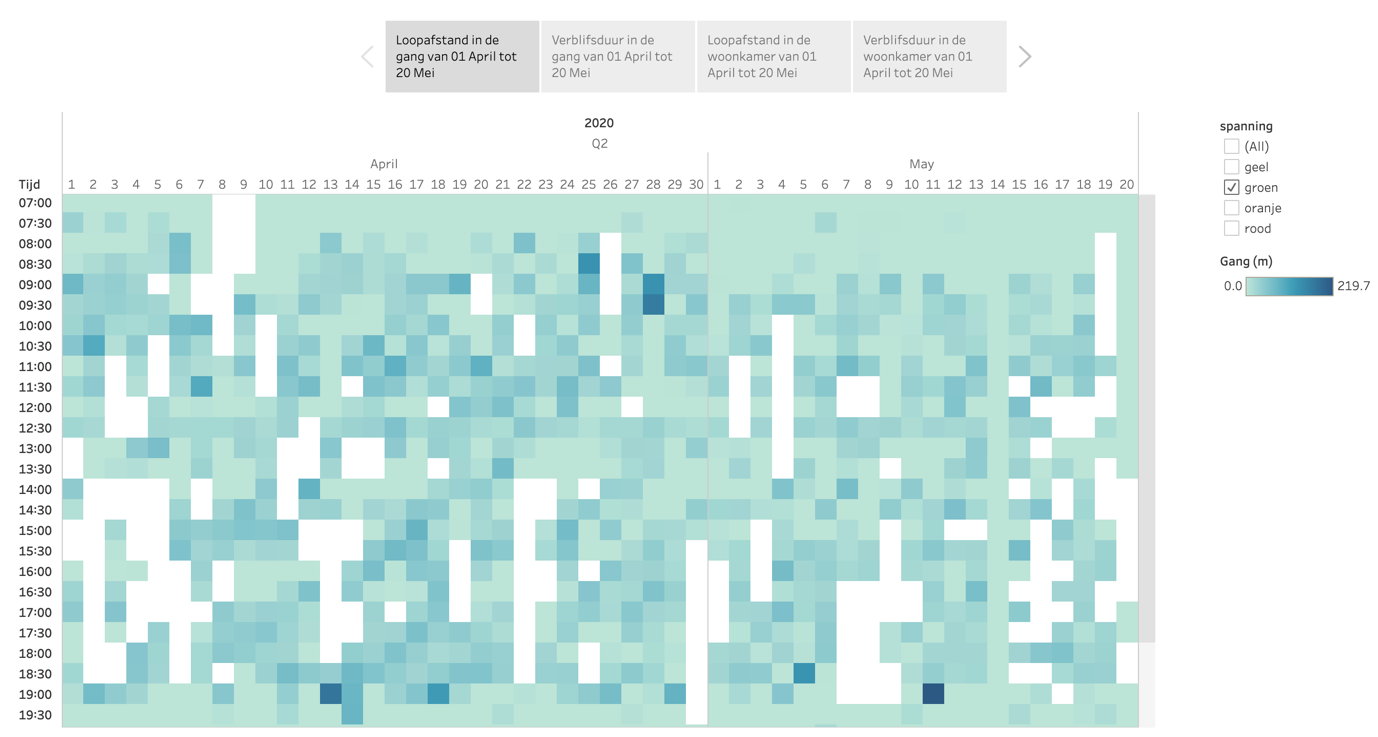


Figure 5. Movement distance in the hallway for Participant 1 superimposed with stress-rating filter (“green” phase selected) from 1^st^ April to 20^th^ May.
