# Supplementary material for: A Digital Platform for Facilitating Personalized Dementia Care in Nursing Homes: Formative Evaluation Study
Source: JMIR Form Res. 2021 May 28;5(5):e25705. doi: 10.2196/25705 (PMC8196358; doi:10.2196/25705)
Supplement: Multimedia Appendix 7 [file formative_v5i5e25705_app7.docx]

# **Multimedia Appendix 7.** Detailed description of thematic analysis results.

# Detailed description of thematic analysis results

## Types of insights

##### Client level

The insights at the client level revolve around what the care team identified for each PwD participant, as mentioned in Table 2, the care team refer to PwD as their clients. First of all, all three caregivers relate the day structure of each PwD with the stress level of the PwD. The key moments mentioned in the day structure are dining time, getting-up time, sleep time, time for going to the toilet. The caregivers think when the day structure was not followed, the PwD was more likely to get agitated. Caregiver 1 described when a PwD gets agitated, caregivers tend to forget the day structure of this PwD. Caregiver 3 commented “it is nice to see my colleagues bring him to his room after lunch”, since this is part of the day structure of this PwD. The doctor also reflected that the tile plots could help her to “identify the day structure a PwD has been having”. The dietitian identified a PwD “goes to sleep much earlier” than she expected from the tile plots. This indicates the digital platform could reveal some insights about the day structure of the PwD.

Moreover, all three caregivers, psychologist and doctor commented on when do unrest moments occur in a day for the PwD participants and what could be the causes. Interestingly, for the same PwD, the psychologist discovered he “walks more in the afternoon when he is agitated” while the doctor identified that there is no relationship between walking distance and stress rating of this PwD. Caregiver 3 postulated that this client walked more around late afternoon because he wants to be helped with going to bed. The dietitian mentioned that if the walking distance is high during the meal time, it could be that the client is “in stress”, and probably “did not eat”. This highlights the care team could identify some insights about the unrest moments of PwD with the digital platform.

The rest of the themes in this cluster are based on insight from one team member. The mapping plots helped caregiver 3 to identify unusual movement trajectories of her client. The doctor noticed that “the connection (between restlessness and stress) is no longer there” for a PwD from the tile plots and then she drew on her experience and postulated that it could be a change in behavior of this PwD as the disease progresses. Caregiver 3 noted that her client is more physically active in the morning than in the afternoon from the mapping plots. Lastly, caregiver 1 noticed that the medication is sometimes not effective for her client from the combined plots.

##### Ward level

The insights at the ward level go beyond the insights at an individual level, and focus more on the ward ambiance. First, it surprised the doctor that when participant 1 was in stress, moving up and down the corridor, the caregivers were not by his side. Caregiver 1 also noticed that sometimes participant 1 is alone in the living room, which should be avoided. This implies the care team is aware of interacting with PwD, and these interactions could be seen from the digital platform. Caregiver 3 pointed out that the mapping plots enable her to “see what other clients and caregivers are doing”, and this helps her to understand the social environment which the client is in. The dietitian also mentioned that she would like to see other PwD when they have interactions with one specific client that she focuses on. The psychologist wondered “I need to find out the reasons for the patterns, I don’t have a conclusion at the moment…or there might be other residents in the ward who have a pattern that influences him.” This underlines that knowing the interactions with other PwD could help the care team to get a more complete overview about the PwD participants, and these interactions can be seen from the digital platform. Lastly, the dietitian found that many PwD are “everywhere” in the ward during eating time from the mapping plots, which might indicate a chaotic dining environment.

##### Team level

The insights at the team level concerns what the care team could improve to facilitate better personalized BPSD management for PwD. Caregiver 3 and the psychologist think the daily reports should be written in more detail. The psychologist expressed that “there is a lack of corresponding daily reports to explain what happened”, when the stress is recorded for the client. Caregiver 3 commented “we need to be more specific in our reports”, since the reports are not detailed enough to “explain the occasions where stress happened” and “explain the stress rating change” most of the time. Together, the digital platform helps the care team to be aware of the importance of detailed reports.

From analyzing the data, all caregivers reflected that they should adhere to the tag usage more, since they noticed how missing data could hinder the data analysis process. They aim to “give the tag directly to the client” when they pick up the tags from the office without carrying the tag and do some other tasks first. The doctor pointed out, from the mapping plots, that the care team walk much more than the PwD. With the same plots, caregiver 1 reflected she walked too fast when she has a lot of tasks to do, which “could cause stress in the clients”, and caregiver 3 stated “it is important to see how my behaviors affect the client”. This reveals that the digital platform could support the care team to understand more about their behaviors and their influence on clients if any. Finally, caregiver 3 found the mapping plots are useful for presenting an overview about their workflow, that is, “all the daily things carried out in the ward”.

## Types of actions

##### Investigation

The actions at investigation level revolve around how the care team plans to answer the questions they have after data analysis. To begin with, the psychologist, doctor, dietitian, and caregiver 3 discovered some insights which they are not certain about the reasons behind. They indicated that they would like to discuss it with other team members in meetings. For example, the psychologist would like to find out with caregivers why a client is in stress around 3pm; the doctor would like to reflect with caregivers about their long walking distance; the dietitian would like to talk to caregivers to understand why only few clients are sitting near the table during meal time; caregiver 3 plans to discuss with her colleagues to understand the unusual movement trajectory of her client. This indicates some insights generated in data analysis could sometimes trigger questions to be investigated further.

Caregiver 2 and caregiver 3 said they will use the digital platform to monitor if they have followed the day structure of the clients, caregiver 3 also added: “it’s hard for me sometimes to recognize any inconsistency when I do my work”. This implies some insights from the digital platform could help caregivers to investigate if they have followed the day structures of clients.

Caregiver 3 mentioned the day structure of her client has recently been changed, and she would like to evaluate if this change in day structure could be beneficial for her client by examining if there is any change in the stress-rating recording. This suggests some insights from data analysis could help the care team with evaluating the effect of changing care plan on the stress level of PwD.

##### Implementation

The actions at the implementation level represent how the care team plans to implement the insights they are sure about in the care practice. Caregiver 1 put forward a few changes in the day structure of her client after analyzing the data, which are “bring the client to the bathroom twice a day even though he did not ask for it”, “providing him snacks to eat in the afternoon”, “check if he is awake at 9am”. She also plans to discuss with doctor about the insight that antipsychotic drug is not effective for her client, and if changes should be made on medication. The dietitian noticed a PwD sleeps much earlier than she thought, hence she will let the caregivers know to “reduce the snacks to two times” from “three times” in the afternoon. This reveals that some insights from the digital platform could help the care team to update care plans for the clients. Nonetheless, not all members think the care plans of their clients should be changed, caregiver 2 commented the care plan is “already very structured” for her clients, the only thing is the caregivers should follow this day structure.

Next to this, caregiver 1 plans to discuss with her colleagues to ensure at least one caregiver stays in the living room if her client is there, and caregiver 3 decided to make more agreements with the caregivers to “make the day structure rigid” for all clients. The dietitian thinks “there is a lot we can do” for creating a quiet dining environment, and she would like to talk with the caregivers about this. This implies some insights from the digital platform make the caregivers more aware of their working practice (and other team members to be more aware of the working practice of the caregivers), and could possibly lead to an improvement in the care delivery.

Caregiver 3 reflected this analysis helps her to know when her client tends to get tense, and she will be more prepared when this time is reaching during her shift. This suggests that some insights from data analysis could help caregivers to predict when a client is likely to get stressed and the team can prepare accordingly.

## Perceived Usefulness

All caregivers find that they can use data in the digital platform as evidence to support their views in addition to what they have observed and their gut-feelings in meetings. When they are not sure about their understanding of a client, they could check with the data.

The doctor finds the digital platform is useful for her work, she has identified a few questions to be investigated with the caregivers after the data analysis. But with only three case studies, she thinks there is not enough evidence to prove the usefulness of the digital platform.

The psychologist finds the digital platform is an enabling tool for her to triangulate the subjective reports of the caregivers with objective data. Because stress-rating is “quite subjective in itself”, she explained, “maybe when the caregivers are tired, they think clients are in stress more”. She found the tile plots combining stress-rating and location data helps her to interpret the stress-ratings more objectively.

The dietitian finds the tile plots of total walking distance helpful because she can estimate the calories burned by a client, and from the same plot, she can deduce the day structure of this client. The mapping plots help her to have an impression about the dining environment in the ward. The dietitian thinks the digital platform will be more useful if there is more data about food.

The manager finds the digital platform not helpful for her work, because she cannot find the answers to her questions. For example, one of her question is about how to allocate the tasks to the caregivers. She mentioned an overview is needed, which includes not only about the interaction time between caregivers and a client, but also what the caregivers and the client are doing. She explained “the caregivers have more duties to do than only to give care to the clients”. But she thinks if the clients are “in bed or sitting alone in the living room”, then the caregivers should do something about these situations. The manager thinks the digital platform cannot help her with balancing the time for the caregivers on interacting with clients and the time on other tasks. When a few insights generated by the care team were mentioned to the manager, she expressed these are the things that she already knew, and therefore, the digital platform did not add any value to her work.

## Areas for improvement

##### Data collection

The manager mentioned that she is interested to know “what are the effective methods of managing BPSD for each client” from the digital platform, thus would like see data describing “what has been done to reduce the stress” in a client, and all three caregivers admit it is “time-consuming to write detailed reports”, and caregiver 3 wonder if there is any chance to collect “data on body language”, which she could use to decide if a client is in stress and what caregivers have done. The dietitian would like to see more data on “how much food has been offered” for the digital platform to be more relevant to her work. This indicates more data types could add value to the digital platform.

The psychologist pointed out that the digital platform will reveal more insights when the data is collected for a new client, whom they do not know much about, and the manager suggested that collecting data for PwD with physical restlessness will be more insightful. This highlights the importance of deciding whose data to be collected for the digital platform to be useful.

The doctor raised an issue on the discrepancy in the subjective data by pointing out that the stress rating and daily reports sometimes contradict each other: “I don’t know if it is because they (the caregivers) find it is difficult to estimate the stress in the residents, or the stress rating system is not clear to them, or they simply find the stress-rating form is hard to fill in.”

##### Data visualization

The psychologist would like to see the report when she “hovers on the data of that day”, and all caregivers and the doctor expressed similar opinion towards seeing the according daily report without consciously selecting them from a filter. This underlines the importance of reducing unnecessary clicking, hence cognitive workload, to integrate the quantitative data and qualitative data closer together in the visuals. Caregiver 1 expressed that she would like to see how many times her client “moves back and forth in a day” given the location data collected, this is because this movement pattern is an indication of this PwD in high stress. This implies sometimes parameters specific to the observed behavioral patterns of a PwD could be added to the digital platform for improved insight gathering. The dietitian would like to know “what the clients are doing at times when they should eat”, to do so, she would like a filter which enables her to focus on the eating times during a day. This suggests filtering data by time could be efficient for users to zoom into the time frame they are interested in.

##### Data analysis

The long analysis time has been mentioned a few times in the interviews. Caregiver 1 and caregiver 2 mentioned the data analysis process is time-consuming, specifically, caregiver 1 spent 1.5 hours in analyzing the data which is “too long”, caregiver 2 indicated “it is very difficult to look at the data when caring for clients”. The doctor mentioned “the data are interesting, and it is just an investment of time to look at it”. The psychologist, on the other hand, “do not mind take some time for the analysis”, and it took her half an hour to go through all the visualizations. The dietitian also commented “it is not time-consuming and I understand what it is”. This opens up the design direction to reduce analysis time for users to ensure all members of the care team will not be hesitant about using the digital platform.

Members of the care team have different opinions on who should analyze the data. The manager reported that data analysis is cumbersome according to some caregivers, while caregiver 3 reflected there are “thinkers and doers” in the team. The doers “do not want to look at the data because they think their time is for the clients”, and the thinkers “like looking at the data and give guidance to the doers”. This raises the question of who should do the data analysis with the digital platform.

Caregiver 1 was not certain about the insights she obtained are correct and wondered “what the other caregivers would think about the data”. Psychologist suggested to assign a team member to analyze the data first, and then bring the insights to the regular meeting and discuss with other members. Within the meeting, members will reflect on if they see the same insights, and discuss what are the reasons behind it. This draws attention to the importance of a workflow in data analysis for the digital platform to better fit with the working routine of the care team. Lastly, caregiver 1 inquired about the possibility of receiving some insights generated by the digital platform itself, which coincides with the future vision of the digital platform, that is, facilitating the care team with data analysis by sending notifications with algorithm-derived insights. In this way, the analysis time, as discussed above, could possibly be shortened.
